# Quantitative Serum NMR Spectroscopy Stratifies COVID-19 Patients and Sheds Light on Interfaces of Host Metabolism and the Immune Response with Cytokines and Clinical Parameters

**DOI:** 10.3390/metabo12121277

**Published:** 2022-12-16

**Authors:** Titus Rössler, Georgy Berezhnoy, Yogesh Singh, Claire Cannet, Tony Reinsperger, Hartmut Schäfer, Manfred Spraul, Manfred Kneilling, Uta Merle, Christoph Trautwein

**Affiliations:** 1 Werner Siemens Imaging Center, Department for Preclinical Imaging and Radiopharmacy, Eberhard Karls University Tübingen, 72076 Tübingen, Germany; 2 Institute of Medical Genetics & Applied Genomics, University Hospital Tübingen, 72076 Tübingen, Germany; 3 Bruker BioSpin GmbH, Applied Industrial and Clinical Division, 76275 Ettlingen, Germany; 4 Department of Dermatology, Eberhard Karls University Tübingen, 72076 Tübingen, Germany; 5 Cluster of Excellence iFIT (EXC 2180) “Image-guided and Functionally Instructed Tumor Therapies”, Medical Faculty, Eberhard Karls University Tübingen, 72076 Tübingen, Germany; 6 Department of Internal Medicine IV, University Hospital Heidelberg, 69120 Heidelberg, Germany

**Keywords:** metabolomics, lipoproteins, SARS-CoV-2, inflammation, glycoprotein, biomarker

## Abstract

The complex manifestations of COVID-19 are still not fully decoded on the molecular level. We combined quantitative the nuclear magnetic resonance (NMR) spectroscopy serum analysis of metabolites, lipoproteins and inflammation markers with clinical parameters and a targeted cytokine panel to characterize COVID-19 in a large (534 patient samples, 305 controls) outpatient cohort of recently tested PCR-positive patients. The COVID-19 cohort consisted of patients who were predominantly in the initial phase of the disease and mostly exhibited a milder disease course. Concerning the metabolic profiles of SARS-CoV-2-infected patients, we identified markers of oxidative stress and a severe dysregulation of energy metabolism. NMR markers, such as phenylalanine, inflammatory glycoproteins (Glyc) and their ratio with the previously reported supramolecular phospholipid composite (Glyc/SPC), showed a predictive power comparable to laboratory parameters such as C-reactive protein (CRP) or ferritin. We demonstrated interfaces between the metabolism and the immune system, e.g., we could trace an interleukin (IL-6)-induced transformation of a high-density lipoprotein (HDL) to a pro-inflammatory actor. Finally, we showed that metadata such as age, sex and constitution (e.g., body mass index, BMI) need to be considered when exploring new biomarkers and that adding NMR parameters to existing diagnoses expands the diagnostic toolbox for patient stratification and personalized medicine.

## 1. Introduction

Almost three years after the first infection with a novel virus, which shortly after was entitled “Severe Acute Respiratory Syndrome-Coronavirus 2” (SARS-CoV-2), the impact of the resulting pandemic is slowly decreasing, although Coronavirus Disease 2019 (COVID-19) will remain part of everyday life in the foreseeable future [1]. Although SARS-CoV-2 and the resulting pandemic became an early focus of research from a wide range of disciplines, the resulting disease is still not fully understood. Among a large variety of genetic and immunology-based investigations, the use of quantitative nuclear magnetic resonance (NMR) spectroscopy, along with other techniques such as mass spectrometry, offers the chance to obtain novel insights into the specific alterations and disturbances of the metabolisms of infected persons and might be a helpful tool for deciphering the disease–host response on a molecular level [2]. Furthermore, the successful application of such analytical instruments might play a pivotal role in combating future (infectious or non-infectious) diseases and advancing the field of personalized medicine [3,4,5].

### 1.1. Metabolomics in COVID-19 Research

Previous NMR metabolomics research has shown that COVID-19 is associated with specific and multifaceted changes in the metabolite and lipid profiles of infected patients [6,7], highlighting the disease’s systemic implications. The metabolic fingerprint of COVID-19 in serum is characterized by a disturbance in amino acid metabolism, which is, among other parameters, expressed by decreased levels of glutamine [8,9,10]. On the other hand, increases in phenylalanine and glutamic acid have also been detected [8,9,10,11]. Regarding other amino acids, such as the branched-chain amino acids (BCAAs) leucine, isoleucine and valine, reports are less consistent, especially when considering results based on mass spectroscopy. Some studies have reported elevated levels [12,13], whereas another stated decreased concentrations, with an accompanying decrease in the so-called Fischer’s ratio (BCAAs divided by the sum of phenylalanine and tyrosine) [14]. Sporadic changes in the concentrations of threonine and N, N-dimethylglycine [15], as well as histidine, lysine, tyrosine and ornithine [10], have also been described.

Apart from this, high blood glucose (hyperglycemia) [10,12,14], which was also found with techniques other than NMR [16], low levels of citric acid [15,17] and an elevation in ketone bodies [10,15] seem to be consequences of viral infection. Furthermore, the effects of COVID-19 infection include a decrease in trimethylamine-N-oxide (TMAO) and an elevation in pyruvic acid [10] as well as in succinic acid [10,15].

The lipid profile of SARS-CoV-2-infected patients has been reported to consist most predominantly of reduced levels of Apolipoproteins A1 and A2 [14,18,19], which are exclusive to high-density lipoprotein (HDL) and increased amounts of very low-density lipoprotein (VLDL) and intermediate-density lipoprotein (IDL) Apolipoprotein B [6,19]. Additionally, levels of triglycerides (TG) in blood serum are elevated [6]. In contrast, decreases in serum cholesterol (CH) levels have become apparent [6,10]. This decline is particularly pronounced in HDL-4, the HDL with the highest density [8,10,13,14]. On the other hand, TG levels have been shown to be elevated exceptionally in large low-density lipoprotein (LDL-1, -2) and VLDL [7,10].

### 1.2. Usage of Novel NMR Markers

In addition to quantifying the above-mentioned parameters, NMR spectroscopy also allows for the determination of values for the composite biomarkers of glycoproteins (Glyc), which have been shown to be valuable inflammation markers [20]. The GlycA signal is more prominent and originates mainly from N-acetylglucosamine residues on proteins such as α1-acid glycoprotein [21], to which an immunological function is attributed [22]. Measuring Glyc parameters has become increasingly relevant in inflammatory disorders in recent years [21].

The application of an IVDr (in vitro diagnostic research-only) system provides full, validated and standardized NMR clinical research and screening [23,24,25]. Within a standardized IVDr NMR analysis, the Glyc signal is composed of two peaks, GlycA and GlycB, which represent different glycosylated amino sugar residues on acute phase reactants [26]. Soon after the pandemic’s onset, these inflammatory markers were investigated in blood serum concerning their association with COVID-19 [14]. Another recent finding was the discovery of the supramolecular phospholipid composite (SPC) signal, which derives from choline headgroups of HDL and LDL phospholipids [27]. They are markedly decreased in SARS-CoV-2-infected individuals [26]. Subsequently, the ratio of Glyc and SPC was proposed to be an independent and highly specific biomarker of acute COVID-19 infection [26].

### 1.3. Immunological Aspects of COVID-19 and Their Link to Metabolomics

Severe cases of COVID-19 are characterized by a dysregulated host immune response [28]. This manifests itself in neutrophilia, lymphopenia, and an exaggerated release of a myriad of different cytokines, predominantly interleukins IL-1, -2, -6, -8, -10, -18, interferon (IFN)-γ, tumor-necrosis-factor (TNF)-α and monocyte chemoattractant protein-1 (MCP-1). This process is frequently referred to as a “cytokine storm” [29,30]. However, cytokine levels during severe COVID-19 infection have been shown to be significantly lower than those in other life-threatening conditions, such as septic shock, acute respiratory distress syndrome (ARDS) or cytokine-release-syndrome (CRS) [31,32]. On the other hand, delayed or reduced CD8^+^ T-cell responses [33,34] and CD4^+^- and CD8^+^ T-cell exhaustion [35] seem to be hallmarks of the disease and are associated with severe COVID-19 infection in combination with higher levels of cytokines [36,37]. Additionally, it was shown that early and robust upregulation of antiviral cytokines such as type I IFNs (IFN-α and IFN-β) and IFN-γ seems to be crucial for general viral clearance [38] and a mild course for the disease [39,40]. These mechanisms seem to be hampered by SARS-CoV-2, resulting in uncoordinated and ineffective immunological responses in severe life-threatening cases [41,42].

The manifestations of COVID-19 are very complex, and host metabolism and the immune system are closely interrelated in a rather complex manner [43]. Furthermore, COVID-19 is a disease in which comorbidities such as diabetes and metabolic syndrome are predisposed to severe disease. This fact has been attributed to insulin resistance in obese and diabetic individuals, enabling a state of low-grade chronic inflammation, in which SARS-CoV-2 can bring derailment [44].

### 1.4. Aims of the Study

Of note, most published NMR metabolomics research draws from its investigations of samples of COVID-19 patients taken during hospitalization or at admission, which provide insights into mainly moderate-to-severe disease courses. Initial comparisons of metabolic profiles of patients differentially affected by the disease have shown interesting results in hospitalized patients [11,18]. We want to build on these results to investigate how transferable the findings mentioned above are to mild-to-moderate disease courses of outpatients and earlier disease stages, and to investigate how they differ from more severe disease courses in our cohort.

In addition, alterations in metabolism can also be attributable to influences related to hospitalization, such as a higher stress level, fasting or changes in diet [45], especially when hospitalized patients receive parenteral nutrition. These variables may need to be considered when investigating the specific metabolic and lipoprotein profiles of the disease.

In this study, we quantified a selection of cytokines and chemokines (targeting inflammation) and combined them with the quantitative NMR spectroscopy of metabolites, lipoproteins and inflammatory markers to correlate them with records from a study in which an ambulatory care model for SARS-CoV-2-infected individuals was tested [46].

A total of 534 blood samples were all collected during home visits, and only 71 out of 329 patients had to be hospitalized during the ongoing course of COVID-19 infection. Additionally, we determined whether the novel NMR markers Glyc and SPC can be of complementary use when interpreting them alongside well-established inflammatory markers such as lymphocyte count, CRP or ferritin [47,48,49]. By utilizing these different layers of information, we aim to gain a deeper pathophysiological understanding of mild-to-moderate courses of COVID-19 infections to enable effective patient stratification.

## 2. Materials and Methods

### 2.1. Patient Recruitment and Sample Collection

Essentially, IVDr-based research in clinical metabolomics enables investigations of observed changes across patient groups while correlating these results with clinical metadata or other findings with a high degree of precision, particularly in the field of NMR spectroscopy [50]. We also think it is feasible to identify potential disease-derived biomarkers using a quantitative method for metabolomics analysis [51].

We performed analyses of 534 serum samples obtained from 334 COVID-19 patients in the acute disease phase who underwent patient care in an ambulatory setting in the Rhein-Neckar/Heidelberg (Germany) area. Blood was drawn once from 194 patients, twice from 80 patients and three times from 60 patients within a couple of days after an initial positive COVID-19 test. For patients who provided multiple samples, sampling was performed within a maximum of 14 days. The study design is described elsewhere in detail [46].

Of note, for this IVDr-based project, three major inclusion and exclusion criteria differ from the cohort investigated by Lim et al. [46].

(1) Only patients with a biobanked serum sample were included in the IVDr cohort;
(2) In the IVDr cohort, 3 patients were included who had been hospitalized prior to study inclusion due to COVID-19 (inclusion of these patients was performed at the release timepoint for subsequent monitoring in an ambulatory setting after hospital release; the first sampling was performed 1, 2 and 6 days after discharge from the hospital and 2, 6 and 7 days after symptom onset);
(3) The IVDr cohort consisted both of patients who entered data into the application(which was used for reports of symptoms and vital parameters) and of patients that didn’t.This allowed patients who were unable to use the app to be included (e.g., due to high age, no mobile device, etc.). Therefore, our IVDr cohort differs to a relevant extent from the published analysis [46], in which only patients with ambulant parameters before hospitalization were included.

The determination of virus variants was not performed. However, based on the data collection period (September 2020–May 2021) and epidemiological reports, it can be assumed that most cases were caused by the wild type and the alpha variant [52]. At the time of sample collection, only 6 of the 329 patients had been vaccinated against SARS-CoV-2.

After quality control (2.2), the analyzed cohorts comprised 155 male and 174 female COVID-19 patients and 305 sex- and age-matched controls. During the disease, 71 subjects (21.6%), 46 males and 25 females, were hospitalized. A detailed description of baseline characteristics can be found in the Supplementary Materials (Table S1).

Blood samples were collected throughout the morning during home visits by a nurse when a clinical indication arose, e.g., worsening of symptoms or lab parameters. Accordingly, because the subjects’ diets were not controlled, it seems unlikely that there is a significant confounding effect as a result of fasting. After collection, blood samples were stored in a Styrofoam box and were transported to the laboratory, where they were centrifuged according to standard operating procedures. Isolated blood sera were frozen at −80 °C and were transported on dry ice to the University Hospital of Tübingen, where they were stored at −80 °C again until analysis. For each blood sample, conventional laboratory parameters commonly used during the care of COVID-19 patients (e.g., (CRP), white blood cell count (WBC) and ferritin) were collected as part of the medical care conducted in Heidelberg. Furthermore, Lim et al., provided a detailed set of metadata, which yield information regarding anthropometrics, medication, age, smoking status and the acute symptoms of the infection. The usage of samples and data was approved by the Ethics Commission of Heidelberg Medical University (S-324/2020), and all participants signed a written informed consent according to the Declaration of Helsinki.

### 2.2. Quantitative NMR Spectroscopy

Raw NMR spectra were acquired using Bruker body fluid B.I. methods [53]. Sample preparation was performed following the included standards of the procedure to ensure reliable results. For quality control, the B.I. BioBankQC^TM^ module was applied. For quantification, the modules B.I. QUANT-PS^TM^ for metabolites and B.I. LISA^TM^ for lipoproteins, respectively, were applied (All B.I. modules: Bruker BioSpin GmbH, Ettlingen, Germany). Blood serum samples were thawed for approximately 30 min at room temperature before 400 µL of each aliquot was pipetted into a 1.5 mL PTFE container and mixed with 400 µL of a commercially prepared pH 7.4 sodium phosphate plasma buffer from Bruker. The mixture was then shaken gently for 1 min before extracting 600 µL of it to fill a Bruker 5 mm NMR tube. The SampleJet cooling setting was set to 279 Kelvin. Monodimensional ^1^H-NMR spectra were acquired using a 5 mm triple resonance (TXI; ^1^H, ^13^C and ^15^N) room temperature probe on a Bruker IVDr Avance III HD 600 MHz system, which was operated using Bruker’s standard NMR software TopSpin (Version 3.6.2, Bruker BioSpin GmbH, Ettlingen, Germany). Five monodimensional ^1^H NMR spectra types were collected for each blood sample with water peak suppression and varied pulse sequences to selectively observe molecular components. First, a NOESY (Nuclear Overhauser Effect SpectroscopY) 32-scan NMR experiment was used to show the NMR spectrum quality (via the B.I. BioBankQC™) and to enable the quantification of metabolites (i.e., glucose, lactic acid and amino acids of the B.I. BioBankQuant-PS™) and high-molecular-weight compounds, such as lipoproteins (as shown in B.I. LISA™). Then, a 32-scan CPMG (Carr–Purcell–Meiboom–Gill, filtering out macromolecular resonance signals) program was run, as well as 32-scan DIFF (DIFFusion measurements of, primarily, macromolecular signal massifs [54]) and 64-scan PGPE (Pulsed Gradient Perfect Echo, used primarily for Glyc and SPC quantification [55]). Moreover, a two-dimensional NMR experiment, 2-scan JRES (J-RESolved spectroscopy), was included with the IVDr methods and was performed to analyze J coupling constants. Additionally, JRES can be useful for manual data look-up and represents a vital piece of the novel Bruker PhenoRisk PACS™ software (Bruker BioSpin GmbH, Ettlingen, Germany). The NMR experiments utilized a group of sample-dependent parameters, like the frequency offset O1 and 90° pulse P1 duration.

All recorded spectra were quantified in full automation. The parameters, GlycA, GlycB and SPC, were subsequently determined with the B.I. PACS^TM^ module. Previously published work has enabled the standardization of the approach used in this project and supports its reliability [26,56]. Exemplary annotations can be found in Supplementary Material (Figure S1).

To obtain a meaningful and high-quality data set, we performed quality control prior to analysis, resulting in the exclusion of 25 samples due to a linewidth of >2.3 Hz. The vast majority of measurements yielded a high ρ, which quantifies the correlation of the calculated fit with the metabolite signal. An overview of this can be found in Supplementary Material (Figure S2). Accordingly, regarding the B.I. QUANT-PS^TM^ module, no cut-off for ρ was defined.

To enable the interpretation of the acquired metabolomics profiles in the serum of COVID-19 patients using B.I. QUANT-PS^TM^ and B.I. LISA^TM^, Bruker BioSpin GmbH kindly provided corresponding serum data of 305 healthy sex- and age-matched individuals (without further information according to donor conditions). Accordingly, in Section 3.1 and Section 3.2, where comparisons are made with healthy controls, we work only with the parameters from these modules.

### 2.3. Quantification of Cytokines

Cytokine analysis was performed on all samples from COVID-19 patients who were found to show a CRP-value > 10 mg/L at some point during acute infection. This preselection process resulted in 309 samples from 171 patients enrolled for testing.

Targeted cytokine and chemokine levels were determined using LEGENDplex Human Inflammation Panel 1 (13-plex) with the V-bottom Plate multiplex assay (#740809; BioLegend). In brief, 15 µL of serum was diluted with 15 µL of assay buffer; from this diluted serum, 25 µL was used for further processing in the assay. Standard curves were prepared with a 4-fold serial dilution method for all cytokines and chemokines (standard with known concentrations) with 7 data points to calculate the actual cytokine and chemokine levels in each sample. Diluted samples (25 µL; 1:1) or standards (25 µL) were added in a 96-well plate followed by 25 µL of assay buffer and 25 µL of capture beads before the plate was incubated at room temperature for 2 h (on a plate shaker). After incubation, the plate was washed, and 25 µL of biotinylated detection antibodies was added to each well, followed by incubation for 1 h. Furthermore, after incubation with the detection antibodies, 25 µL of a streptavidin-phycoerythrin (SA-PE) antibody was added to each well, and the 96-well plate was incubated for an additional 30 min. Finally, the 96-well plate was washed with washing buffer 2 times before 150 µL of wash buffer was added, and the samples were transferred to 5 mL flow cytometry tubes. Each sample was analyzed with a multi-color flow cytometer (FACS BD LRSFortessa, Becton Dickinson, Franklin Lakes, NJ, USA). Flow cytometry files were transferred to analysis software (Data Analysis Software Suite; LEGENDplex cloud-based software), and the final analysis was performed according to the manufacturer’s instructions.

### 2.4. Statistics and Data Illustration

All quantifications that withstood quality control were considered independently in the statistical analysis, so results from patients who provided multiple samples (see Chapter 2.1) were not treated differently. We decided to utilize this procedure because the sample collection was performed when a clinical indication emerged, which suggested the persistence of the acute disease. In addition, the samples were not taken at the exact same time point in the acute disease phase (e.g., the two samples from Patient A could have been taken 5 and 7 days after infection, and those from Patient B could have been taken 8 and 10 days after infection). We aimed to level this effect by considering all samples.

Statistical analysis was performed with the quantified parameters using the web-based tool MetaboAnalyst 5.0 [57]. For all (2 group-) analyses, we excluded all features that showed >50% missing values. The remaining missing values were estimated using feature-wise k-nearest neighbor imputation, which showed robust effects in the context of metabolomics data [58]. To correct for heteroskedasticity, which is not uncommon in this context, as concentration magnitudes from metabolites, lipoproteins and other markers vary strongly, we performed a logarithmic transformation. For univariate analysis, volcano plots were generated, which are combinations of *p*-values generated from unpaired t-tests and fold changes (FC). For figure generation, thresholds for the *p*-value and FC were established at 0.05 and 1.2, respectively. The False Discovery Rate (FDR) was controlled using the Benjamini–Hochberg correction to maximize statistical power [59]. For correlation analyses, we focused on Pearson’s correlation coefficient.

Further analyses were conducted using multivariate approaches of unsupervised principal component analysis (PCA) and supervised orthogonal partial least squares discriminant analysis (OPLS-DA). PCA was performed to identify outliers, simplify the dataset and assess an overview of the variation within. Besides that, OPLS-DA was used to assess the discrimination between two groups and to identify the parameters that drive this separation. MetaboAnalyst’s biomarker toolbox was used for further biomarker analysis [57]. The univariate analysis and the volcano and violin plots were illustrated using GraphPad PRISM 9, and original figures from MetaboAnalyst were used to show the multivariate analyses of PCA and OPLS-DA.

**Figure 1:**
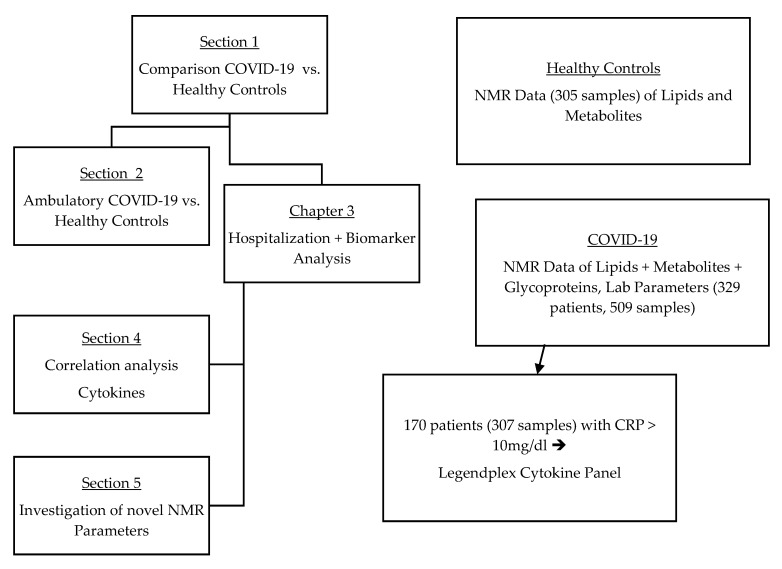
Consort diagram showing an overview of the performed analyses and the available data (after quality control).

The consort diagram (Figure 1) was constructed using Microsoft PowerPoint 2019 MSO (Version 2211, Microsoft, Redmond, WA, USA), and the diagrams illustrating the confounder analysis for the novel NMR markers were designed with Microsoft Excel 2019 (Version 2211, Microsoft, Redmond, WA, USA). The depiction of the correlation analyses was performed using R with modifications with Inkscape. Further editing of the figures was performed using Adobe Acrobat Pro.

## 3. Results

B.I. QUANT-PS^TM^ analysis provided quantified values for trimethylamine-N-oxide, amino acids and derivatives, carboxylic acids, choline as an essential nutrient, keto acids and derivatives, carbohydrates and derivatives, and dimethylsulfone as a sulfone. All the resulting 38 parameters are defined in mmol/L. In addition, the B.I. LISA^TM^ analysis yielded detailed information on the lipid profiles of the investigated samples, including multiple parameters such as cumulative values of triglycerides (TPTG), cholesterol (TPCH) and apolipoproteins (Apo) A1 (TPA1), A2 (TPA2) and B100 (TPAB). From these parameters, the ratios of LDL-cholesterol (LDCH) to HDL-cholesterol (HDCH), which is LDHD, and of Apo-B100 to Apo-A1 (ABA1), were calculated. Regarding TG, CH, free cholesterol (FC) and phospholipids (PL), concentrations are indicated for each of the fractions (HDL, LDL, IDL and VLDL). Moreover, for HDL, LDL, and VLDL, the results are also specified for several subfractions. An increasing density defines this classification from subfractions with a lower number (e.g., H1) to those with a higher number (e.g., H4). The HDL group was divided into four subfractions, the VLDL group was divided into five subfractions and the LDL group was divided into six subfractions. The same principle applies to Apo A1 and Apo A2 for HDL and Apo B100 for VLDL, IDL and LDL. Additionally, the total particle number (TBPN) and the particle numbers of VLDL (VLPN), IDPN (IDPL) and LDL (LDPN) were calculated from the concentration of ApoB100 in the sample. In the case of LDL, the particle numbers are also specified for each subfraction. Besides details about the particle number, which refer to a concentration range of mmol/L, all B.I. LISA^TM^ parameters are defined in mg/dL.

### 3.1. Comparison of COVID-19 vs. Healthy Controls

**Figure 2:**
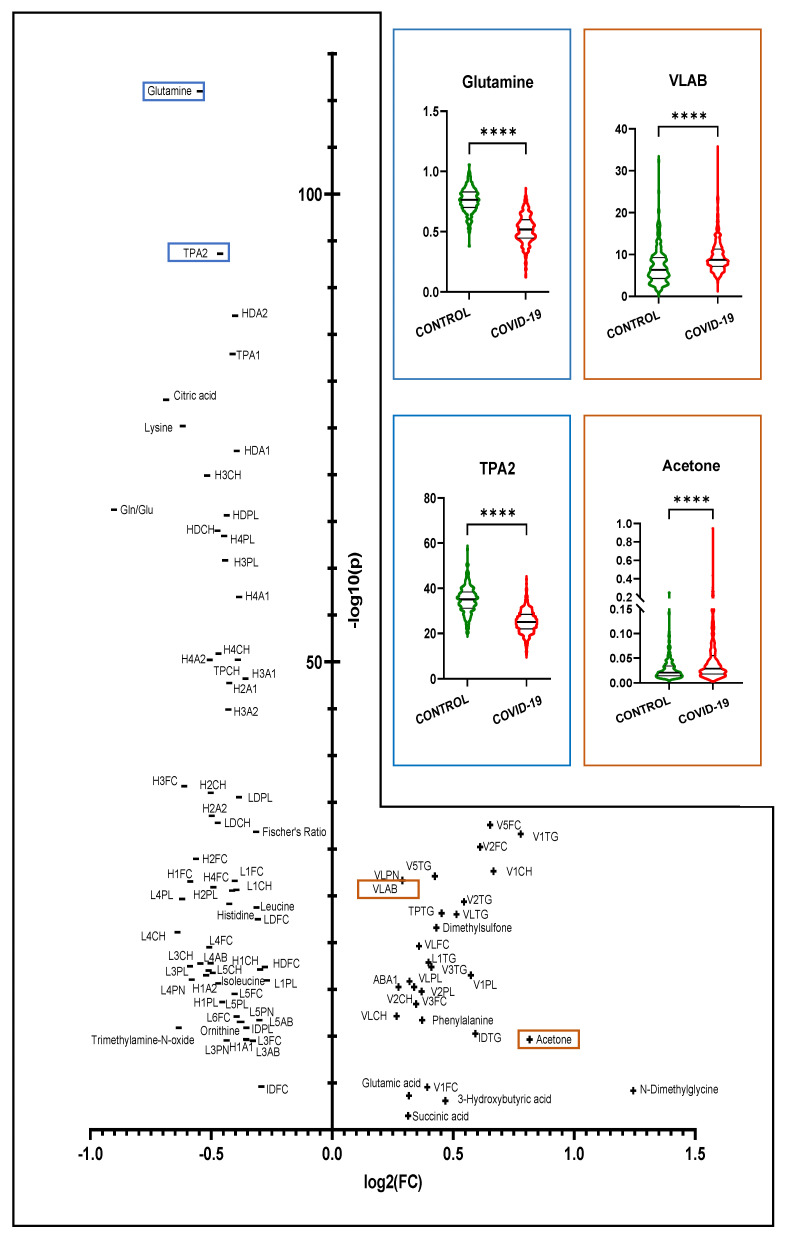
Volcano plot showing the univariate comparison of the whole COVID-19 cohort with healthy controls. The logarithmized fold change (FC > 1.2) is plotted on the X-axis, whereas the Y-axis indicates the significance in the form of the logarithmized *p*-value (FDR; *p* < 0.05). In the upper right, exemplary violin plots show the differences in significantly altered parameters. Four asterisks indicate a *p*-value of <0.0001.

In the serum of SARS-CoV-2-infected individuals, we found a distinctive profile, which includes substantial changes in metabolites and lipoproteins and clear discrimination from the profile of healthy controls. Figure 2 provides the results of the univariate analysis in the form of a volcano plot with 17 significantly changed parameters from the B.I. QUANT-PS^TM^ analysis and 74 significantly altered parameters from the B.I. LISA^TM^ analysis. A positive log_2_ (FC) value on the x-axis indicates upregulation of the corresponding parameter. In contrast, a negative log_2_ (FC) value implies that the substance is found in a lower concentration in COVID-19 patients than in healthy controls. The logarithmized and FDR-adjusted *p*-values (*p* < 0.05) are indicated on the y-axis. Higher concentrations of phenylalanine, N, N-dimethylglycine, glutamic acid, sarcosine, creatine and ketone bodies (acetone, acetoacetic acid and 3-hydroxybutyric acid) were determined in the sera of COVID-19 patients when compared with the sera of healthy controls. In contrast, we measured lower concentrations of glutamine, lysine, citric acid, histidine, leucine, ornithine, trimethylamine-N-oxide and isoleucine upon COVID-19 infection. Furthermore, the glutamine/glutamic acid ratio and the Fischer’s ratio, which quantifies the ratio of branched-chain amino acids (isoleucine, leucine, valine) to aromatic amino acids (phenylalanine, tyrosine), were significantly lower in the sera of COVID-19 patients than in the sera of individuals from the control cohort. Regarding the detailed lipid profiles, we identified an increase in the concentration of apolipoprotein B100 (Apo B) in the VLDL (VLAB) fraction but a decrease in Apo B and in particle numbers of large LDL particles (L3AB, L4AB, L5AB; L3PN, PN and L5PN). Besides that, we observed a strong decrease in apolipoprotein A1 (TPA1) and A2 (TPA2) in general and in all HDL fractions (HDA1, HDA2, H1A1, H1A2, H2A1, H3A2, H4A1 and H4A2), indicating the reduced presence of HDL in the blood of SARS-CoV-2-infected patients. These changes were associated with an increase in the Apo A and Apo B100 (ABA1) ratio. The increase in VLAB can be attributed to a general increase in the VLDL particle number (VLPN). This also explains the increase in the VLDL (VLCH, V1CH and V2CH) cholesterol fractions, although we determined a decrease in the total cholesterol (TPCH) plasma level. The latter was also reflected in a significant decrease in the HDL (HDCH, H1CH, H2CH, H3CH and H4CH) and LDL (LDCH, L1CH, L3CH, L4CH and L5CH) cholesterol levels. The free cholesterol concentrations changed similarly and were reflected by an increase in VLDL (VLFC, V1FC, V2FC V3FC and V5FC) and a decrease in HDL (HDFC, H1FC, H2FC, H3FC and H4FC) and LDL (LDFC, L1FC, L3FC, L4FC, L5FC and L6FC), as well as in IDL (IDFC) concentrations. In contrast, we determined an increased level of total triglycerides (TPTG) in the sera of SARS-CoV-2-infected patients, which was mainly connected to upregulations in VLDL (VLTG, V1TG, V2TG, V3TG and V5TG) and intermediate-density lipoprotein (IDTG) fractions. However, we also determined an increase in the content of TG in the lowest-density LDL (L1TG). In addition, levels of phospholipids were elevated in low-density VLDL (VLPL, V1PL and V2PL), whereas these were decreased in IDL (IDPL), HDL (HDPL, H1PL, H2PL, H3PL and H4PL) and LDL (LDPL, L1PL, L3PL, L4PL and L5PL). An overview of the absolute concentrations of parameters in B.I. QUANT-PS and B.I. LISA in healthy and infected subjects is provided in Table S2. Using the multivariate approaches of PCA and OPLS-DA, no outliers could be detected, and the disparity of the groups could be demonstrated (Figure S3).

### 3.2. Comparison of the Outpatient Cohort vs. Healthy Controls

Next, we conducted identical analyses of the sera of patients who were not hospitalized during the disease but who remained in an ambulatory setting, and we once again compared them with the healthy controls.

**Figure 3:**
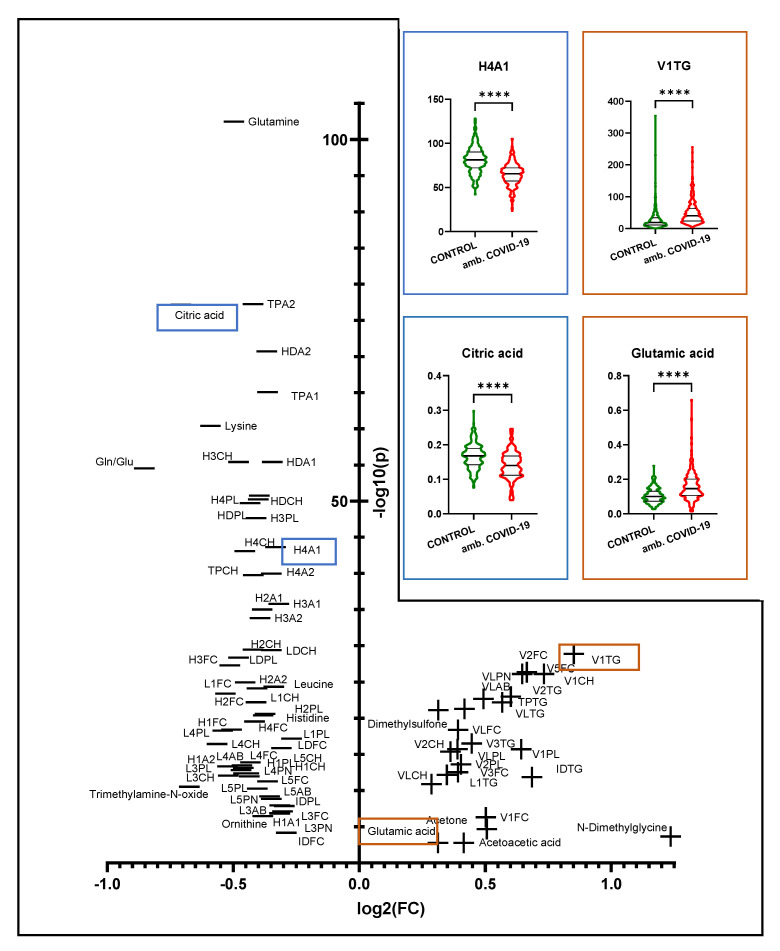
Volcano plot showing the univariate comparison of the COVID-19 patients who were not admitted to the hospital with the healthy controls. The logarithmized fold change (FC > 1.2) is plotted on the X-axis, whereas the Y-axis indicates the significance in the form of the logarithmized *p*-value (FDR *p* < 0.05). In the upper right, exemplary violin plots show the differences in significantly altered parameters. Four asterisks indicate a *p*-value of <0.0001.

We hereby reproduced the described above results to a large extent but also found some observable major differences. Figure 3 indicates that phenylalanine, one of the few compounds that are elevated in hospitalized patients (Figure 2), was not significantly changed in outpatients compared with healthy controls. This also explains why the Fischer’s ratio did not yield a significant change in this comparison. Furthermore, in contrast to hospitalized COVID-19 patients, we determined no increase in succinic acid and ABA1 levels in the sera of ambulatory COVID-19 patients.

In addition, it is noticeable that CH levels in total (TPCH) and HDL (HDCH) were not decreased as strongly as in the analysis of hospitalized COVID-19 patients. In addition, in contrast to hospitalized patients, we were unable to determine significantly altered fractions of free cholesterol, HDFC, LDFC and L6FC in the sera of COVID-19 patients in an ambulatory setting.

As reflected in Figure 3 and the Supplementary Materials (Table S3), the ketone bodies were less elevated in the sera of COVID-19 patients who stayed in home care when compared with the results we obtained from the comparison of the whole COVID-19 cohort with healthy controls (Table S4). In this context, the FC of acetone declines from 1.76 to 1.42. The FC of 3-hydroxybutyric acid falls below our established threshold of 1.2. OPLS-DA showed good discrimination of the two groups, and PCA identified no outliers (Figure S4).

### 3.3. Comparison of Hospitalized Sub-Cohort with the Sub-Cohort That Stayed in Outpatient Care

**Figure 4:**
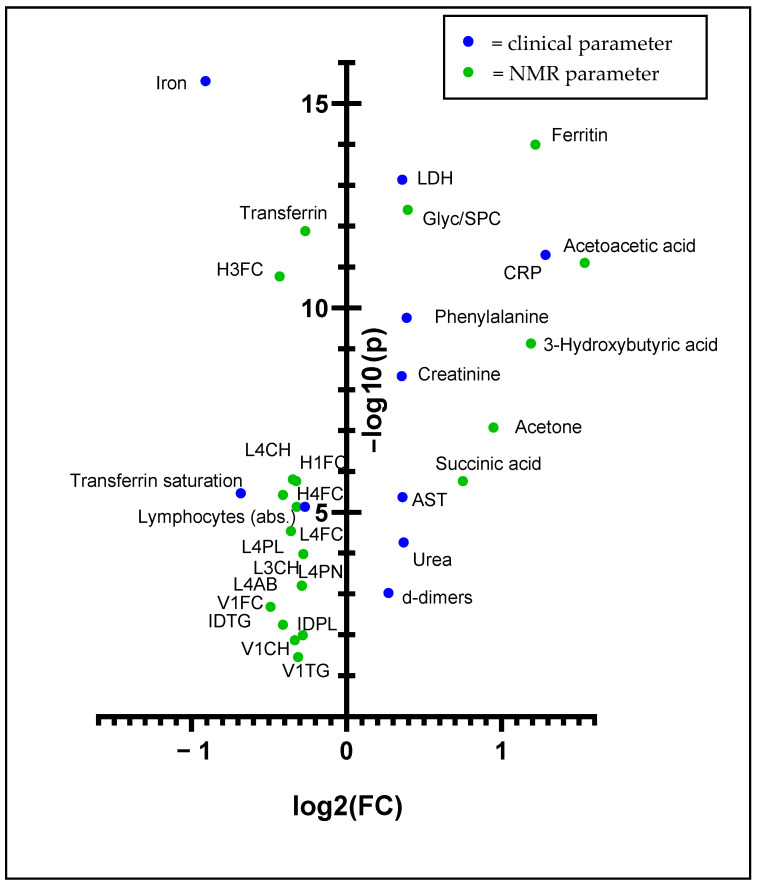
Volcano plot (FDR *p* < 0.05) showing the univariate analysis of the patients who were admitted to the hospital with patients who stayed confined at home. Blue dots indicate clinical parameters, whereas green dots represent IVDr-based NMR parameters.

After comparing COVID-19 patients with healthy individuals, we next focused on more in-depth analyses within the COVID-19 cohort. In addition to the NMR data on lipoproteins and metabolites, we included laboratory parameters from routine clinical practice as well as cytokine values. We used these data to compare the profiles of patients hospitalized at some point before or after sample acquisition and the subjects that stayed at home. In this context, it is important to emphasize that the sampling of hospitalized patients was still exclusively performed during home visits, in most cases before an admission occurred and rarely after hospitalization. Because we already have shown the comparisons of the whole COVID-19 cohort and exclusively of the patients who stayed in home isolation with the healthy controls, some of the results are redundant. Nonetheless, we aimed to show how the NMR parameters from B.I. QUANT-PS^TM^ and B.I. LISA^TM^ can be combined with the novel B.I. PACS^TM^ module and the clinical parameters. Figure 4 represents the results of this analysis.

**Figure 5:**
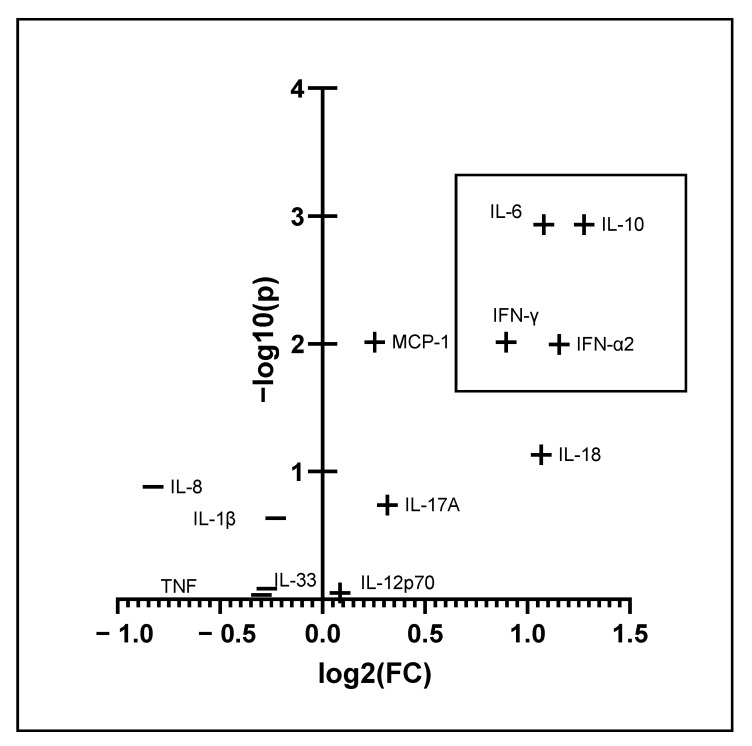
Volcano plot showing the univariate analysis of cytokines in patients who were admitted to the hospital against patients who stayed confined at home. The black rectangle on the right encloses the cytokines that showed significance (FDR *p* < 0.05) and a fold change of more than 1.2.

Figure 5 displays the analysis of cytokine levels in the sera of SARS-CoV-2-infected patients. Most importantly, we determined cytokine levels exclusively in the sera of patients with a CRP > 10 mg/L at some point during the disease.

Although the two compared groups both consisted of COVID-19 patients, several significantly changed parameters were particularly noticeable. As illustrated in Figure 4, the ketone bodies acetoacetic acid and acetone were elevated in patients who exhibited COVID-19 more severely. 3-Hydroxybutyric acid also revealed an increase just below our FC threshold of 1.2 in hospitalized patients, as shown in Supplementary Material (Table S5). Besides that, phenylalanine and succinic acid were increased in this regard. Likewise, the clinical laboratory parameters ferritin, LDH, CRP, creatinine, AST, urea and d-dimers were increased in COVID-19 patients. A closer inspection of the plot indicates that the Glyc-to-SPC ratio increased when compared with the ambulatory COVID-19 cohort. Interestingly, acetoacetic acid did not emerge significantly in the first analysis of COVID-19 patients against healthy controls, although a fold change of 1.98 was determined.

Several features regarding the lipoprotein profiles were significantly altered between the two investigated groups. Interestingly, IDTG, V1FC, V1CH and V1TG were downregulated in the sera of more severe COVID-19 cases compared with milder cases. In the primary comparison (Section 3.1.), we determined elevations of these parameters exclusively in COVID-19 patients but not in healthy individuals. Besides that, significant decreases can be observed in the denser subfractions of LDL (L4CH, L4FC, L4PL, L4AB, L4PN, L3CH and L3PL) and HDL (H4FC, H3FC, H1FC and H1A2). Complementarily, we determined downregulations of the clinical blood concentrations of iron, transferrin, transferrin saturation and lymphocyte count.

Because many more men (*n* = 46) than women (*n* = 25) were hospitalized, we aimed to identify a potential bias by repeating the comparison of hospitalized patients against patients who stayed in an ambulatory setting for the acute disease course for the two sexes separately. The relevant tables are displayed in the Supplementary Materials (Tables S6 and S7). Essential features are evident in both analyses, including upregulations of 3-hydroxybutyric acid, Glyc/SPC, phenylalanine, CRP, ferritin and LDH and downregulations in iron levels in the sera of COVID-19 patients. Many of the parameters that showed significance (FDR < 0.05) and an FC of >1.2 in only one comparison yielded uniform changes in the other group but fell below our FC threshold or fell victim to FDR. This was evident for transferrin and lymphocyte count, which were significant in the comparison of hospitalized females against female patients who remained in ambulatory care. This was also evident for acetone, Fischer’s ratio, H4A1, H4CH, H4FC, H4PL, L3CH, creatinine and transferrin saturation in the analysis of hospitalized males against male COVID-19 patients who stayed in an outpatient setting. Nonetheless, some evident discrepancies became visible by comparing the two analyses: AST and GGT were only elevated in the comparison of female COVID-19 patients, and changes in choline (down) and succinic acid (up) were exclusively identified in the comparison of male patients. Furthermore, downregulations in the lipoprotein profile, e.g., in H4A2, VLFC, VLTG and TPTG, only became apparent in the comparison of male patients. It is apparent from Figure 5 that, regarding the cytokine profile, we found significant upregulations in IFN-α2, IL-10, IL-18, IFN-γ and IL-6 in the sera of COVID-19 patients when compared with samples of patients who were hospitalized during their COVID-19 disease course (at some point before or after sample obtainment).

Besides sex, age was another predisposing factor for hospitalization, as hospitalized patients were older (mean age 59.1 yrs. to 53.5 yrs). To address this potential bias, we performed a sex-specific stratification of the patient cohort with age (two strata, threshold: 60 years). Details can be found in the Supplementary Materials (Tables S6 and S7). The prominent findings of this stratification were that ferritin was not significantly elevated in hospitalized male patients under the age of 60, and Glyc/SPC (FC = 1.19; FDR *p* = 0.26) and iron were not significantly altered in female patients under the age of 60 years. Furthermore, phenylalanine showed significant alterations in males >60 years but remained under the fold change threshold of 1.2. H4A1 showed significance in males and females but not in the sub-analyses of female patients. These results should be interpreted cautiously due to the changing cohort sizes.

### 3.4. Biomarker Analysis

**Figure 6:**
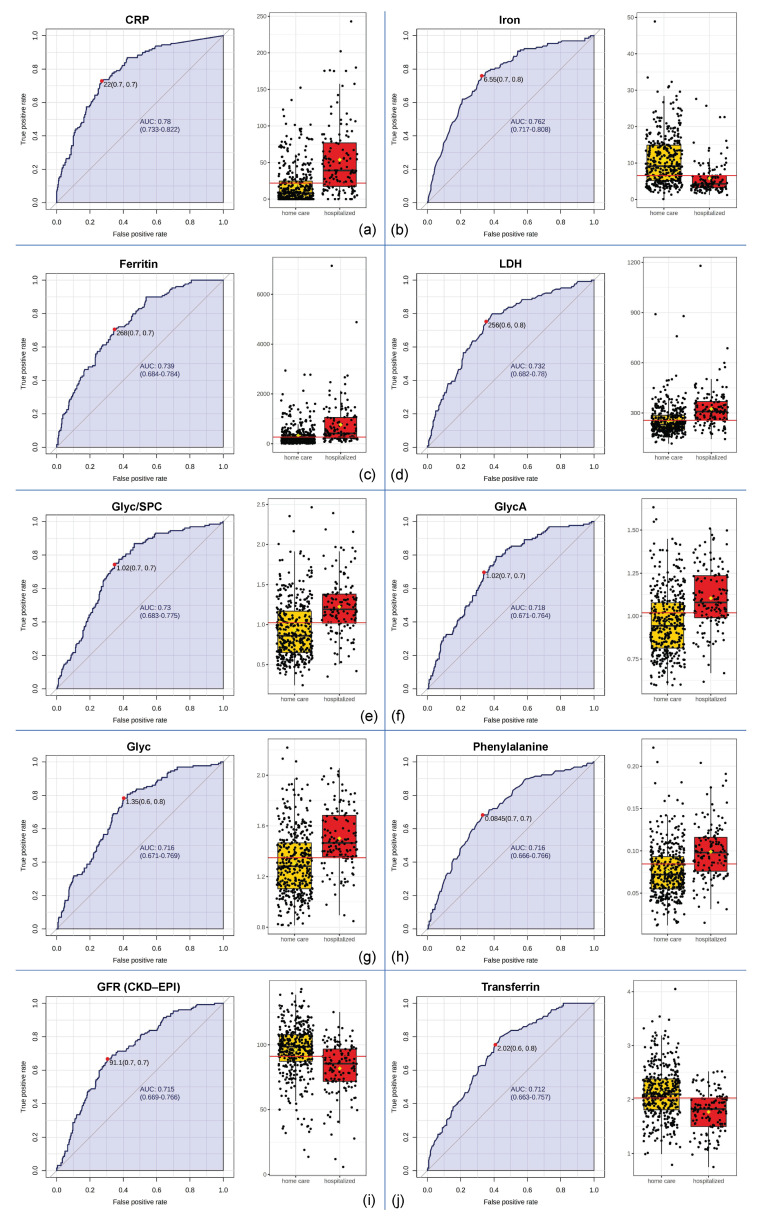
Top ten parameters established from univariate biomarker analysis for hospitalization. For each biomarker, a comparison of the individual values of the respective parameters is shown on the right, and receiver operating characteristic (ROC) curves are found on the left. ROC curves illustrate the number of true positives and false positives for each possible threshold, and the trade-off of these values is quantified in the area under the curve (AUC). An AUC of 0.5 implies a random distribution with no association of the parameter with, in this case, hospitalization, whereas an AUC of 1 implies a perfect predictive model. The parameters are sorted by AUC, with the top parameter can be found under (**a**) and the AUC decreases steadily until (**j**). The red dots on the curves indicate the ideal classification thresholds. For example, an iron threshold of 6.55 (subfigure (**b**)) can correctly identify 70% of the hospitalized COVID-19 patients in our cohort and can correctly classify 80% of the patients who were not admitted to the hospital.

To compare the prognostic abilities and applicability of the NMR-based parameters with the values obtained from the standard clinical laboratory, we performed several univariate biomarker analyses, which are displayed in Figure 6. When evaluating the performance of a biomarker, the area under the receiver characteristic curve (AUROC) is an often-used measure. Here, we summarize the relationships between true positives and false positives for each possible classification threshold. An area under the ROC curve (AUC) of 0.5 implies no association of the parameter with hospitalization, whereas an AUC of 1 implies a perfect predictive parameter. As indicated in Figure 6a, an increase in CRP yielded the strongest performance associated with hospitalization in our cohort (AUC = 0.78), closely followed by iron (AUC = 0.762; Figure 6b), ferritin (AUC = 0.739; Figure 6c) and LDH (AUC = 0.732; Figure 6d). Increases in the NMR-based parameters Glyc/SPC (AUC = 0.73; Figure 6e), GlycA (AUC = 0.718; Figure 6f), Glyc (AUC = 0.716; Figure 6g) and phenylalanine (AUC = 0.716; Figure 6h) exhibited a similar performance to the compared clinical laboratory parameters. Furthermore, it is worth mentioning that HDA1 and H4A1 showed decent predictive abilities, with an AUC of 0.712 and an AUC of 0.703, respectively. Details can be found in the Supplementary Materials (Table S8).

### 3.5. Cytokine Correlation Analysis

**Figure 7:**
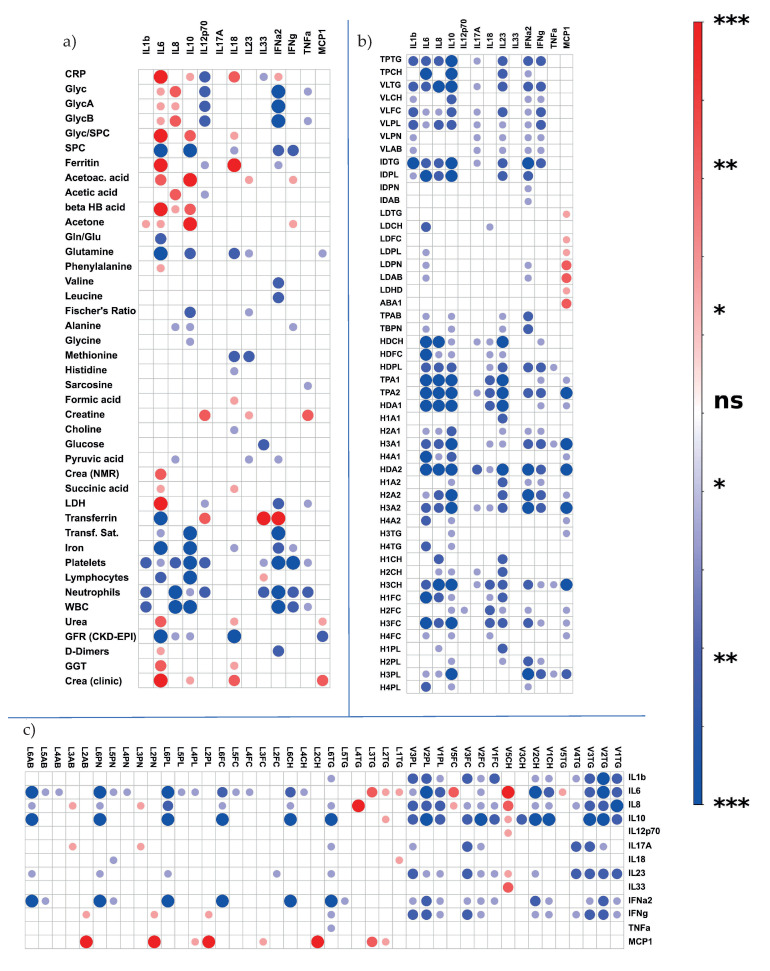
Analysis of correlations between different NMR and clinical parameters and cytokines. The different significance levels are color-coded (see legend on the right). Red dots indicate a positive correlation, and blue dots indicate a negative correlation. One asterisk implies a *p*-value of <0.05, two asterisks are equivalent to a *p*-value of <0.01 and three asterisks indicate a *p*-value of <0.001. On the left side, under (**a**), the figure shows the correlations of the measured cytokines with acute phase proteins, Glyc and SPC, ketone bodies, amino acids and other parameters from B.I. QUANT-PS™, as well as parameters from the clinical laboratory. On the right, under (**b**), the figure shows the correlations of the cytokines with the main parameters from the lipid fraction, as well as with all the markers from the HDL subfraction. The correlations of cytokines with the remaining markers from the other lipid subfractions are displayed under (**c**). A list of used abbreviations can be found in the Supplementary Materials (Table S9).

Next, we focused on the correlation analysis of serum cytokines with clinical and NMR parameters, as shown in Figure 7. The analysis is based on a comparison of the COVID-19 patients, as the control cohort did not include such information, and no remaining serum aliquots were available. In addition, we would regardless expect minimal or no detectable cytokine concentrations in the sera of healthy individuals.

In our comparison of hospitalized patients with patients who stayed in an ambulatory setting, IL-6, IL-10, IFN-γ and IFN-α2 emerged as markers of a more severe course of COVID-19 infection. Figure 7a shows correlations of increased IL-6 and IL-8 values with the values of the acute-phase proteins CRP and ferritin, Glyc and ketone bodies. Glyc levels correlated negatively with IFN-α2 levels, whereas SPC exhibited exclusively negative correlations with all determined cytokines. Glutamine, one of our cohort’s most explicit markers for COVID-19 infection, yielded highly significant negative correlations with IL-6 and IL-10 levels. In addition, we found correlations of cytokines with clinical laboratory parameters. For example, increased IL-6 values correlated positively with lactate dehydrogenase (LDH) values, a cell death marker, and negatively with iron, lymphocyte count and glomerular filtration rate (GFR). Figure 7b shows that monocyte chemoattractant protein-1 (MCP1) represents the only cytokine in our analysis that correlates positively with the main parameters from the lipid fraction or HDL parameters. Strikingly, TPTG, despite its increase in COVID-19, shows only negative correlations with the increasing cytokines IL-6 and IL-10, which are prominent in our hospitalization analysis. Significant negative correlations were also found between apolipoproteins A1 and A2 and various cytokines. IL-6 correlated negatively with ApoA1 of HDL in the sera of our COVID-19 patient cohort. This was more prominent in dense HDL (H4A1) than that in less dense subfractions. The least dense HDL (H1A1) subfraction showed no correlation with IL-6. The remaining correlations of cytokines with lipid subfractions are shown in Figure 6c. Interestingly, IL-6, IL-10 and IFN-α2 showed highly significant negative correlations with the parameters in high-density LDL (LDL6). Moreover, there was a positive correlation of IL-6, IL-8 and IL-10 with V5CH and V5FC, and there were negative correlations with V2CH and the remaining VLDL parameters. The longitudinal trajectories of IL-6 and IFN-γ for patients who were hospitalized and for patients who cured themselves at home can be found in the Supplementary Materials (Figure S5). Interestingly, the levels of IFN-α2 and IFN-γ decreased steadily in patients who remained in outpatient care. IFN-γ was barely detectable the third sampling time. In hospitalized patients, the levels remained elevated.

### 3.6. Investigation of Novel NMR-Based Inflammation Parameters in the Context of COVID-19

**Figure 8:**
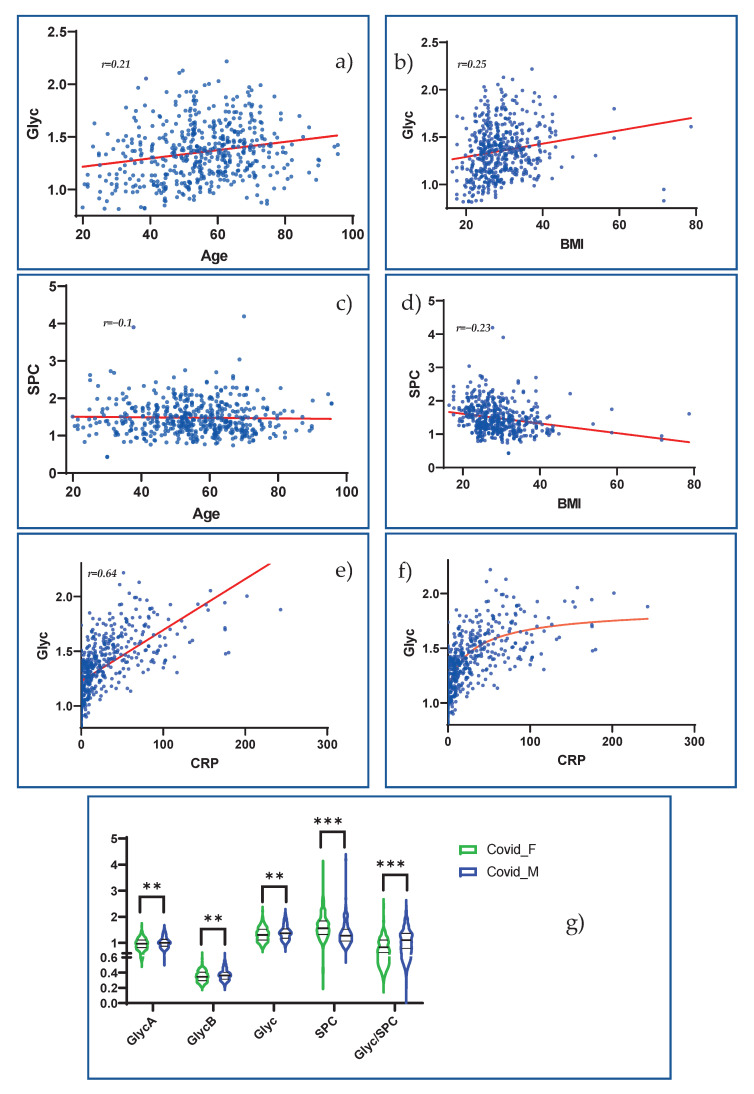
Relationship of 1H-NMR inflammation markers Glyc (sum of glycoprotein A and B) and SPC with possible confounders of age (**a**,**c**) and BMI (**b**,**d**). In subfigures (**e**,**f**), we examined the correlation of Glyc with CRP. The red line, which represents the trend line in subfigures (**a**–**f**), represents a linear correlation in subfigure (**e**) and a nonlinear correlation in subfigure (**f**). Correlation coefficients (Pearson) are given in the upper left corner of the subfigures with an analysis of linear correlation. (**g**) A possible correlation also exists between Glyc and SPC with the respective sex. Asterisks describe the significance level of a sex-specific distinction (** = *p* ≤ 0.01, **** = *p* ≤ 0.0001).

Finally, we addressed the potential susceptibility of the NMR inflammatory markers Glyc and SPC to age, BMI and sex, which we identified as potential confounders. As indicated in Figure 8, BMI showed a positive correlation with Glyc (Figure 8b) r = 0.25) and a negative correlation with SPC (Figure 8d) r = −0.21). In contrast, older age seemed to be only markedly associated with higher Glyc values (Figure 8a) r = 0.21) but not with strongly altered SPC values (Figure 8c) r = −0.1). Sex influences also appeared probable when looking at Figure 8a, as Glyc and especially SPC were found in higher values in males. These effects seemed to accumulate in the Glyc/SPC ratio. Additionally, we correlated Glyc to CRP to approximate the inflammatory significance of this parameter, and we found a strong correlation with a Pearson’s r of 0.64. (Figure 8e). As shown in Figure 8c, we tested the application of a nonlinear trend line, which seems to represent the relationship between CRP and Glyc better.

## 4. Discussion

Using different commercial quantitative NMR spectroscopy modules and a targeted cytokines & chemokines assay, we compared the metabolic, lipoprotein and inflammatory profiles of a large cohort of SARS-CoV-2-infected individuals alongside more than 300 healthy control samples. Although 71 participants were administered to the hospital during their disease course, our results mainly characterize the phenotype of a mild-to-moderate COVID-19 disease. Adding clinical parameters to the analysis, we discriminated patients who were hospitalized from those who cured themselves in an ambulatory setting. Of note, changes in clinical laboratory parameters must be viewed cautiously, because many hospital admissions were presumably conducted due to pathological changes in these parameters [46]. To further characterize metabolic, lipoprotein and inflammatory disruptions in COVID-19, we first compared our data to previously reported results and then linked them to our determined cytokine values.

### 4.1. Abnormalities in Energy and Amino Acid Metabolism Might Indicate Viral Intervention and Host Response and Might Have Immunological Implications

Interestingly, one of the frequently highlighted findings in studies of hospitalized patients, which we could not reproduce in our analyses, is hyperglycemia. Hyperglycemia might play an essential role in the development of COVID-19, as diabetes mellitus (DM), besides obesity and arterial hypertension, has been identified as an apparent risk factor for infection and a severe disease course [60,61]. Additionally, hyperglycemia and poor glycemic control are independently associated with severe infections [62] and a worse outcome in COVID-19 patients [16,63,64]. Furthermore, the question of whether SARS-CoV-2 can trigger the onset of DM in apparently healthy individuals was raised [65]. As mechanisms driving this development, direct pancreatic destruction by the virus [66] and persistent influences of strongly elevated proinflammatory cytokines [67,68] were discussed. In our comparison of hospitalized patients with patients who stayed in an ambulatory setting (Section 3.3), we found a tendency toward discretely elevated glucose levels (FC 1.08, FDR 0.06) (data not shown in Results section). We hypothesize that hyperglycemia may be a feature of advanced disease rather than mild-to-moderate progression. Supporting this notion, Correia et al., also observed increased glucose levels with increased disease severity [11]. On the other hand, a mildly altered glucose metabolism might be masked by increased consumption due to the mechanisms described below.

Hyperglycemia was reported to favor SARS-CoV-2 thriving in infected monocytes from bronchoalveolar lavage in vitro by sustaining increased glycolysis levels in aerobic situations [69]. The authors attributed this upregulation of aerobic glycolysis to the effects of hypoxia-inducible factor 1-α (HIF-1 α), a transcription factor stabilized by hypoxia [9], and reactive oxygen species (ROS). The production of ROS in the mitochondria was found to be elevated in SARS-CoV-2-infected monocytes, and treatment with antioxidants (to neutralize ROS) was found to inhibit HIF-1α stabilization, viral replication and IL-1β expression [69]. These findings support the idea of a switch in metabolism similar to the Warburg effect [9,70]. The Warburg effect was first described in cancer research and refers to a high level of glycolysis despite sufficient oxygen levels, which can typically enable the usage of oxidative phosphorylation. This might be a product of the viral intervention itself or a result of present hypoxia [9].

The Warburg effect can lead to decreased introduction of pyruvate into the mitochondria and thus into the Krebs cycle, and it can lead to increased activation of the enzyme citrate lyase [70]. This downregulation of the Krebs cycle might be indicated by a decrease in citric acid [71], as observed in our cohort and as commonly observed in other studies [17,18]. Moreover, other substrates for the Krebs cycle seem to accumulate, as other research has found elevated levels of pyruvate and α-ketoglutarate [11,72]. We did not determine upregulation in pyruvate, which might be due to our cohort’s overall milder disease severity, as pyruvate has been proposed as a potential predictor for a severe disease course [72]. Inhibition of the Krebs cycle might favor increased fatty acid biosynthesis via an excess of acetyl-CoA, the results of which might be reflected in the lipid profiles we observed.

In this context, decreases in leucine, isoleucine, histidine and lysine might be signs of general amino acid catabolism to compensate for dysregulated energy metabolism [9]. The elevation of α-ketoglutarate, described by Ceperuelo et al., might be a result of accelerated glutamine metabolism [70,72], besides skeletal muscle breakdown [14]. This might be influenced by hypoxia and could explain elevations in glutamic acid as a byproduct of this process [9]. Independently from why glutamine levels are so downregulated, it likely has relevant consequences for the course of the disease [73,74]. Glutamine is an essential nutrient for immune cells [75], which might implicate immunosuppression due to worse functionality and the degradation of lymphocytes in cases of glutamine deprivation [76], leading to COVID-19 aggravation.

Supporting the theory of increased amino acid catabolism, we found decreased levels of ornithine. This non-proteinogenic amino acid functions as an essential player in the urea cycle to dispose of excess of nitrogen, which results from amino acid catabolism. Indeed, in our study, urea levels were elevated; however, in conjunction with elevated creatinine levels, we see this more as a sign of kidney function impairment.

Such metabolic reprogramming has been linked to the promotion of viral replication [70] and might have further immunological implications, because SARS-CoV-2-infected monocytes were shown to express higher levels of glycolysis, pro-inflammatory cytokines and hypoxia (HIF-1α expression) which leads to overall T-cell exhaustion [69], and impaired CD4^+^- and CD8^+^ T-cell proliferation. Additionally, the Warburg effect might promote the activation of neutrophils and pro-inflammatory M1 macrophages, whereas anti-inflammatory M2 macrophages seem to rely on oxidative phosphorylation [70]. We hypothesize that metabolic reprogramming may trigger or exacerbate immune response derailment in this manner. Icard et al., also stated that the Warburg effect leads to various effects such as the promotion of micro-vessel thrombosis, which could explain the elevation in d-dimers in COVID-19.

Interestingly, we observed signs of disturbed iron metabolism in the group that was hospitalized (low levels of iron (hypoferremia), low transferrin, low transferrin saturation, high levels of ferritin and an average count of red blood cells and hemoglobin), which is in line with the findings we published previously [77]. Because ferritin and transferrin are parts of the acute-phase reaction, their alterations should be seen with caution in inflammation. SARS-CoV-2 was postulated to rely on iron uptake from hemoglobin to produce ROS to protect itself from elimination by the immune system [78]. The resulting ROS might link these findings to the development of the postulated Warburg effect in COVID-19 [70]. Because we found no difference in the amount of hemoglobin in the comparison of hospitalized patients with outpatients, we hypothesize that this mechanism, if present, is only reflected in a mild form in this cohort. The predictive value of hypoferremia in COVID-19 is not only apparent in this study but was also discussed in a study whose cohort did not overlap with the population in this investigation [77]. A higher presence of ROS in COVID-19 might also be a valid explanation for rises in phenylalanine, which has been linked to extensive immune activation in ovarian cell carcinoma [79]. The authors of that study hypothesized the impairment of 5,6,7,8-tetrahydrobiopterin, an essential cofactor in the conversion of phenylalanine to tyrosine, due to oxidation by ROS. It was suggested that these ROS might be induced by various cytokines, such as IFN-γ or IFN-α2 [80]. Because we only detected a correlation between phenylalanine and IL-6, which both behave stage-dependently in a similar manner, we consider a viral influence likely.

More importantly, in this study, SARS-CoV-2 infection was also found to cause an elevation in the ketone bodies 3-hydroxybutyric acid, acetoacetic acid and acetone, which discriminated hospitalized patients from those who stayed in ambulatory care. Generally, an elevation in ketone bodies indicates a catabolic state, which can be seen as a mechanism employed by the host to battle a viral infection [81]. Of note, because samples were obtained from individuals confined at home, in which their diets were not controlled, we consider an influence by fasting unlikely. Instead, we conclude that the observed increase in ketone bodies might be an expression of the disease, as stated before by Bruzzone et al. [10].

The immunological relevance of ketone bodies in the context of viral infections was discussed previously in general [82]. As growing evidence suggests that SARS-CoV-2 hampers the synthesis and release of ketone bodies, which restricts potential alternative energy sources for immune cells [83], even interventional ketone body diets or supplementation have been discussed as countermeasures against COVID-19 [84]. Interestingly, it has been shown that the attenuated functionality of CD8^+^ T-cells obtained from SARS-CoV-2-infected individuals [33,34,36] can be improved by a ketogenic diet [85]. We found highly significant correlations between IL-6 and IL-10 with acetoacetic acid, 3-hydroxybutyric acid and acetone. Thus, we hypothesize that increases in ketone bodies might be a part of immunological host responses, either mediated or accompanied by a rise in inflammatory cytokines. Augmented ketogenesis might be enabled by an excess of acetyl-CoA in the liver, which results from upregulated adipose tissue lipolysis, which is common during the acute phase response in infection [86] to increase VLDL packaging and secretion. Increases in VLDL and the TG fraction of IDL—which develops from the decomposition of VLDL in the blood circulation, as observed in our cohort—may be related to this. Besides this, we determined a decrease in Fischer’s ratio, which resulted from elevations in aromatic amino acids (phenylalanine and tyrosine) and decreases in BCAAs. This finding has previously been interpreted as a sign of liver impairment [14], a finding that is typical in COVID-19 [87,88] and that is attributed partly to systemic inflammation and to the influence of IL-6 [89].

### 4.2. Profound Lipoprotein Alterations Might Be Attributable to Effects of the Virus, and Pro-Inflammatory Effects of IL-6 Are Reflected in Specific HDL Profiles

Like other groups, we reported an association of COVID-19 with severe disruption in the lipoprotein profiles of infected individuals [6,10,13,14,90]. In addition to the before-mentioned elevations in VLDL and IDL, one of our key findings was a substantial elevation in the total amount of TG and a strong decrease in the total amount of cholesterol in plasma, which was more pronounced in hospitalized patients. Interestingly, the total amount of triglycerides (TPTG) only showed negative correlations with cytokines, despite both TPTG and IL-6, for example, rising strongly in COVID-19. We hypothesize that the elevation in TPTG might be primarily attributed to the viral intervention to fuel itself and less to the host response. Hereby, a more detailed analysis sheds light on alterations in the fraction of HDL: ApoA1 and ApoA2 were both strongly downregulated (FC 0.76), and cholesterol in HDL showed an even stronger decrease (FC 0.72) with relative upregulation in free cholesterol (FC 0.82). HDL particles were enriched with TG, as the absolute amount did not change compared with the healthy controls. These findings are consistent with the work of other groups with hospitalized COVID-19 patients [7,13]. HDL, which originally exerts beneficial antioxidant and anti-inflammatory properties, can take on a proinflammatory role when the functionality of ApoA1 is impaired during general inflammation with a chronic, acute-phase reaction [91]. This is the case in metabolic disorders such as diabetes, where CH is increasingly transferred from HDL to other lipoproteins with the opposing transfer of TG [92]. Our study’s composition of HDL raises the question of whether HDL in COVID-19 is concordantly altered into a dysfunctional and proinflammatory agent, as these particles show the same profile that we demonstrated in our cohort [92]. A recent study using mass spectrometry to investigate the composition of HDL reported elevations of the acute-phase proteins α1-antitrypsin and serum amyloid A in addition to decreased ApoA1. The authors suggested that ApoA1 is either less synthesized by the liver or is replaced by serum amyloid A, and they demonstrated decreased anti-inflammatory activity [93]. This conversion was attributed to the influence of proinflammatory cytokines, such as IL-6 [93]. In our correlation analysis, we found negative associations of IL-6 with ApoA1 especially but also with other HDL parameters, confirming similar results reported before [7]. In our investigation, this correlation was particularly present in the subfraction of dense HDL (H4A1). The prominence of HDL4 makes sense, because individual particles decrease in density due to the intake of TG and the release of cholesterol. At this point, it should be emphasized that downregulations in H4A1 were found to be independent of sex in our study. This is noteworthy, because SARS-CoV-2-infected men in our study exhibited more substantial changes in their lipoprotein profile than in that of women. This imbalance was reported before [94].

We showed that TG-rich and CH-poor lipid profiles are also reflected in the fraction of LDL. We found decreases in a wide variety of parameters except for TG content, which was elevated in large LDL (L1TG) and unaltered; thus, relatively upregulated in the other subfractions. Interestingly, our correlation analysis found uniform negative correlations of IL-6 (*p*-value in correlation with L6TG only 0.051), IFN-α-2 and especially IL-10 with all the parameters of dense LDL (LDL6). IL-10 has been causally linked to increasing the macrophage intake of HDL and LDL [95], which might be another reason explaining the complex interactions of inflammatory and metabolic profiles.

### 4.3. Several Clinical Parameters and Cytokines Indicate More Severe Disease Courses

Besides the metabolic and lipoprotein profiles of COVID-19, we had a closer look at the NMR-based inflammation markers Glyc and SPC and how they might be able to predict hospitalization in an ambulatory cohort. Indeed, some standard clinical and cytokine parameters have been assigned prognostic values before by indicating an increased amount of inflammation or incipient organ damage. Several of these parameters, such as d-dimers, CRP, ferritin, LDH, lymphocyte count and elevations in IL-6 and IL-10 [47,96], also stood out in our hospitalization analysis. In addition to IL-6 and IL-10, we found relatively elevated levels of IFN-α 2 and IFN-γ in hospitalized patients. A closer look at the development of IFN-α2 (S13) in patients who gave a longitudinal set of three samples revealed that this cytokine showed a decrease in patients who stayed at home.In contrast, it stayed at similar levels in hospitalized patients. This results in overall elevation in IFN-α2 in hospitalized patients, demonstrating the ineffectiveness of this IFN-I response in severe cases, as reported before [41]. IFN-γ showed similar development, as its expression nearly diminished in the samples from the third time point in ambulatory patients. Moreover, it was still high in hospitalized patients, indicating a prolonged immune response.

### 4.4. Pre-Existing Conditions and Obesity Complicate the Identification of Possible Biomarkers

It is worth illuminating that our cohort of 329 COVID-19 patients included 134 patients with pre-existing arterial hypertension. A total of 34 of these patients were additionally already diagnosed with diabetes mellitus. This must be interpreted together with the fact that 68.4% of the patients in our cohort showed a BMI > 25 kg/m^2^, and 35.9% even exhibited obesity with a BMI > 30 kg/m^2^. On average, 54% of adults in Germany are overweight (BMI > 25), and 18.1% exhibit obesity [97]. This could act as a confounder in comparison to healthy individuals, especially when evaluating the altered lipoprotein profile we found in SARS-CoV-2-infected subjects. In our hospitalization analyses (Section 3.3), the difference in BMI between hospitalized and outpatients was only moderate (mean: 29.0 vs. 28.5). However, it should be noted that the cohorts differed slightly in age (mean: 59.1 years vs. 53.4 years) and comorbidities. Of the 71 hospitalized patients, 33 were already diagnosed with arterial hypertension (46.5%), and 101 out of the 258 patients (39.1%) who remained in outpatient care during the whole acute disease phase exhibited this condition. Considering our results and related studies, we postulate that the lipid constellation of specifically altered HDL composition and high TG in COVID-19 presents itself in a similar manner to the profile of metabolic syndrome [92]. The boundaries appear to blur in some places, as preexisting conditions, such as DM or obesity and COVID-19, appear to aggravate each other, not only causing a higher risk in the acute phase of the disease, but also causing persistent limitations in quality of life in the form of long-COVID-19 [98]. This statement does not discount the significance of our and other groups’ findings, as several NMR-based parameters were shown to be reliable indicators of infection and predictive markers of a more severe course with hospitalization.

### 4.5. NMR Biomarkers Perform Well in Stratifying COVID-19 Patients

In the following, we discuss the NMR parameters of Glyc/SPC ratio, GlycA, TPA1/HDA1 and phenylalanine, because these parameters showed similar prognostic capabilities to the clinical parameters of CRP, LDH and ferritin. Of note, regarding the biomarker analysis, it is essential to keep in mind that we are comparing sub-cohorts of the COVID-19 cohort (as NMR inflammatory parameters, cytokines and clinical laboratory values were not available for the control samples); hence, very high AUCs, with strong predictive power (approximately AUC > 0.85), were not to be expected. In addition, it is worth noting that the discussed NMR parameters are not exclusively informative concerning COVID-19, as most of them have also been associated with several other conditions.

Firstly, we want to highlight the potential clinical relevance of phenylalanine. This marker showed significant elevations in the comparison of all COVID-19 patients with the healthy controls (Section 3.1.) with a determined fold change of 1.29 (1.34 in females and 1.27 in males). In the corresponding analysis of ambulatory patients and the healthy controls (Section 3.2.), phenylalanine showed slight but highly significant elevations. It showed strong upregulations in the analysis of hospitalization and good prognostic values in the biomarker analysis, with an AUC of 0.716. Phenylalanine was associated before with increased mortality in patients with acute respiratory distress syndrome [99] and was found to be elevated stage-dependently in patients with ovarian carcinoma, as mentioned before [79]. Additionally, the relationship between phenylalanine and COVID-19 was investigated by Luporini et al., who found the parameter to be a robust marker of disease severity [100], which has similarly been seen in two recent studies [11,18]. The relationship between phenylalanine and immune system activation is underlined by the correlation we demonstrated with IL-6, which is consistent with results from the literature [19]. Apolipoprotein A1 in the HDL fraction (HDA1) showed an AUC of 0.712 in our biomarker analysis. Interestingly, apolipoprotein A1 in the H4 subfraction (H4A1) performed slightly worse, with an AUC of 0.703. As already discussed, we suspect an ongoing transformation of HDL to a proinflammatory and dysfunctional phenotype that is present in the early phase of COVID-19 and in mild-to-moderate disease, respectively.

### 4.6. Glyc and SPC Reliably Indicate COVID-19 Severity and Offer Exciting Possibilities for the Assessment of Inflammatory Activity

Finally, we want to discuss the NMR markers Glyc and SPC in more detail. GlycA was found to possess predictive value in the treatment of HIV [101] and rheumatoid arthritis [102]. It shows elevations in *lupus erythematodes* [103] and enables the tracking of disease severity in inflammatory bowel disease [104], where it reflects mucosal recovery. Because it is primarily understood as an inflammatory marker, GlycA was related to CRP and partly also to IL-6 in the context of diabetes mellitus [105] and in prediction models of cardiovascular incidents [106], where it proved to have considerable expressive power. GlycA is also associated with metabolic syndrome, obesity and insulin resistance [107,108]. Because the NMR signal of GlycA arises from modifications in the glycan branches of glycoproteins that are known as acute-phase reactants [21], it is not surprising that we found correlations of Glyc with CRP (Pearson’s R 0.64) and with IL-6, which mediates the acute-phase response [109]. Interestingly, we found a more significant correlation of IL-6 with the Glyc/SPC ratio (Pearson’s R 0.24, *p*-val < 0.0001) than with Glyc directly. However, the potential value of Glyc should not be underestimated and limited to its relationship with other parameters, such as CRP [20]. Ritchie et al. (2015) showed that GlycA elevations are often chronic and indicate low-grade chronic inflammation in the sense of elevated cytokine levels and increased neutrophil activity. Furthermore, it was used to predict the risk of severe (respiratory) infections in a large population-based study of apparently healthy individuals [110]. Moreover, in an Estonian-population-based study, α1-glycoprotein, the main contributor to the GlycA signal, emerged as a predictor for death from all causes [111]. In relation to the current pandemic, an association of pre-existing elevated Glyc levels with severe COVID-19 courses was demonstrated [112]. Additionally, an increase in Glyc with disease severity was previously demonstrated by Ghini et al. [18].

As mentioned before, SPC, a newly discovered NMR marker derived from LDL and HDL subfractions [113], shows additional power in identifying SARS-CoV-2-infected individuals. We demonstrated the prognostic value of the Glyc/SPC ratio in stratifying COVID-19 patients, as it showed significantly higher values in hospitalized patients compared with those who cured themselves at home, and it showed an AUC of 0.73 in our biomarker analysis. Interestingly, the ratio of Glyc/SPC also shows elevations in the post-acute phase of COVID-19, making it a compelling marker for post-acute COVID-19 syndrome [26]. To assess the susceptibility of these parameters to possible confounders, we correlated Glyc and SPC with BMI and age and checked for eventual sex-specific differences. We were able to show that a higher BMI is associated with higher Glyc and lower SPC, and higher age is primarily associated with higher Glyc. We demonstrated sex-specific differences in these parameters in our cohort, including higher SPC in females and higher Glyc in males. These effects accumulate when the Glyc/SPC ratio is formed. It is difficult to say whether different reference values must be established for different sexes and for different BMI or ages. However it must be considered that immune mechanisms are affected by obesity, old age and male sex, because they constitute risk factors for a severe disease course in COVID-19 [114]. At this point, we must state that our cohort was composed of more men than women with a severe course (hospitalization). Despite sex- and age-specific influences, Glyc/SPC showed significant changes in the comparison of hospitalized patients against outpatients in men over 60 years of age, in men under 60 years of age and in women over 60 years of age. In females under the age of 60, Glyc/SPC was not elevated in hospitalized patients, but because only 17 patients were admitted to the hospital, this result must be seen with caution.

In summary, we postulate that Glyc, independently and in combination with SPC, shows exciting potential both in and outside COVID-19 research, which makes further validation and investigation in the coming years necessary. These steps might enable future translation into clinical practice and can help achieve progress in the field of personalized medicine. From a practical point of view, this seems more feasible than ever, because it has been demonstrated lately that the determination procedure works reliably not only with cost-extensive high-field spectrometers but also with low-field NMR benchtop systems [55].

## 5. Conclusions

From the results of our study, we can infer that COVID-19 is inextricably associated with specific changes in immunometabolism. We largely reproduced the metabolic profile of COVID-19 characterized by other groups through studies of hospitalized patients, demonstrating that most findings are indeed an expression of the disease and do not necessarily include factors predisposing to a severe course. This metabolic and lipid profile is characterized by the extensive dysregulation of energy metabolism, in which we recognize similarities to the Warburg effect and specific changes in lipid profiles that resemble metabolic dysfunction. COVID-19 seems to blur the lines between metabolically healthy and diseased individuals, so further research is necessary to determine if some of the observed alterations are attributable to derailments of pre-existing or newly onset metabolic conditions and not to COVID-19 itself. We used the determined cytokines to uncover possible interfaces of metabolism and the immune system on which quantitative NMR spectroscopy had previously shed light. Finally, we investigated the novel NMR parameters Glyc and SPC. We look forward to seeing what role these markers will play in the characterization of post-acute COVID-19 syndrome and other inflammatory and metabolic diseases, as well as in their use in the field of personalized medicine and precision diagnostics.

## Data Availability

The data presented in this study are available upon request from the corresponding authors due to privacy or ethical restrictions.

## Supplementary Materials

The following supporting information can be downloaded at https://www.mdpi.com/article/10.3390/metabo12121277/s1: Table S1: Baseline characteristics, Table S2: Absolute concentrations of B.I. QUANT-PS and B.I. LISA in the COVID-19 cohort and healthy controls, Table S3: Univariate analysis of ambulatory COVID-19 vs. healthy controls, Table S4: Univariate analysis of COVID-19 vs. healthy controls, Table S5: Univariate analysis of hospitalized sub-cohort vs. outpatient COVID-19 cohort, Table S6: Univariate analysis of hospitalized male sub-cohort vs. male outpatient sub-cohort, Table S7: Univariate analysis of hospitalized female sub-cohort vs. female outpatient sub-cohort, Table S8: Hospitalization Biomarker Analysis, Table S9: Lists of abbreviations and units; Figure S1: One-dimensional ^1^H NMR spectral data of one of the blood analyte solutions, Figure S2: Overview of the ρ-values in BI-QUANT-PS^TM^, Figure S3: Multivariate analysis of COVID-19 vs. control; PCA and OPLS-DA, Figure S4: Multivariate analysis; PCA and OPLS-DA, Figure S5. Box plots of cytokine trajectory.
