# Supplementary material for: Quantitative Serum NMR Spectroscopy Stratifies COVID-19 Patients and Sheds Light on Interfaces of Host Metabolism and the Immune Response with Cytokines and Clinical Parameters
Source: Metabolites. 2022 Dec 16;12(12):1277. doi: 10.3390/metabo12121277 (PMC9781847; doi:10.3390/metabo12121277)
Supplement: Supplementary file 1 [file metabolites-12-01277-s001.zip › metabolites-2000373-supplementary.pdf]

Supplementary Material

Quantitative Serum NMR Spectroscopy Stratifies COVID-19 Patients and Sheds Light on Interfaces of Host Metabolism and the Immune Response with Cytokines and associated Clinical Parameters

Titus Rössler [1], Georgy Berezhnoy [1], Yogesh Singh [2], Claire Cannet [3], Tony Reinsperger [3], Hartmut Schäfer [3], Manfred Spraul [3], Manfred Kneilling [1,4,5], Uta Merle [6,†] and Christoph Trautwein [1,*,†]

1 Werner Siemens Imaging Center, Department for Preclinical Imaging and Radiopharmacy, Eberhard Karls University Tübingen, 72076 Tübingen, Germany
2 Institute of Medical Genetics & Applied Genomics, University Hospital Tübingen, 72076 Tübingen, Germany
3 Bruker BioSpin GmbH, Applied Industrial and Clinical Division, 76275 Ettlingen, Germany
4 Department of Dermatology, Eberhard Karls University Tübingen, 72076 Tübingen, Germany
5 Cluster of Excellence iFIT (EXC 2180) "Image-guided and Functionally Instructed Tumor Therapies", Medical Faculty, Eberhard Karls University Tübingen, 72076 Tübingen, Germany
6 Department of Internal Medicine IV, University Hospital Heidelberg, 69120 Heidelberg, Germany
* Correspondence: christoph.trautwein@med.uni-tuebingen.de
† These authors contributed equally to this work.

Table S1. Baseline Characteristics

Clinical Metadata and Symptoms

Sex	Male	Female	Overall
Participants	155	174	329
BMI>25 kg/m²	123/153 (80.4%)	100/173 (57.3%)	223/326 (68.4%)
BMI>30 kg/m²	60/153 (39.2%)	57/173 (32.9%)	117/326 (35.9%)

	**Mean**	**Median**	**1st and 3rd Quartile**
Age	54.5	54.7	44.5-64.7
BMI	28.6 (males: 29.5; females 27.8)	27.8	24.4-32
max dyspnea (grading 1-5 (mild to severe)) (n=304)	1.77	1	1-2
max breathing rate [/min] (n=281)	20.1	19	16-23

	**Yes**	**No**	**unknown**
Smoking	28	281	20
Hospitalization	71 (46 males, 25 females)	242	0
Oxygen demand (in hospitalized patients)	48 (33 males, 15 females)	23	0
Intensive Care Unit (ICU) (in hospitalized patients)	8 (5 males, 3 females)	63	0
death	5 (2 males, 3 females)	324	0
shivering	7	319	3
limb pain	49	275	5
fatigue	9	315	5
cough	59	265	5
loss of appetite	72	252	5
nausea	28	296	5
vomit	3	318	8
headache	53	271	5
tiredness	79	245	5
impairment of general	68	256	5

well-being			
impairment of drinking	13	311	5
loss of smell or taste	88	236	5
night sweats	35	288	6
dizziness	20	304	5
chest pain	15	309	5
diarrhea	17	307	5
sore throat	16	308	5
palpitations	7	316	6
rhinitis	0	328	1

Pre-existing conditions and medication

	**Yes**	**No**
Previous medical conditions:		
hypertension	134	195
diabetes mellitus	44	285
asthma	44	285
chronic obstructive pulmonary disease (COPD)	12	317
obstructive sleep apnea syndrome (OSAS)	22	307
depression	23	306
coronary artery disease	33	296
cancer (active)	15	314
cancer in past	23	306
stroke in past	16	313
rheumatoid arthritis	10	319
cardiac insufficiency	6	323
hypothyreodism	46	283
inflammatory bowel disease (IBD)	6	323
thrombosis in the past	27	302
transplantation in the past	3	326
pregnancy	4	325
Regular medications:		
beta-blockers	61	268
Calcium-antagonists	32	297
Angiotensin-converting enzyme (ACE)-inhibitors	43	286
Angiotensin II receptor (AT_1)-antagonists	62	267
L-thyroxine	41	288
acetylsalicylic acid	46	283
clopidogrel	5	324

citalopram	13	316
mirtazapine	3	326
venlafaxine	2	327
opipramol/ amitryptiline	5	324
pantoprazole	40	289
statins	46	283
metformin	20	309
allopurinol	14	315
insulin	9	320
oral antidiabetics	10	319
direct oral anticoagulants (DOAC)	17	312
cortisone derivatives	7	322
adalimumab	2	327
azathioprine	1	328
tacrolimus	3	326
rituximab	1	328
methotrexate	5	324

Table S2. Absolute Concentrations of B.I. QUANT-PS and B.I. LISA in the COVID cohort and the Healthy Controls

B.I. QUANT-PS Parameter (mmol/L)	Mean Value in COVID cohort (n=509)	Standard Deviation in COVID cohort	Mean Value in Healthy Control cohort (n=305)	Standard Deviation in Healthy Control cohort
Ethanol	0.18133	0.84152	0.40770	0.36511
Trimethylamine-N-oxide	0.01934	0.01801	0.03069	0.02589
2-Aminobutyric acid	0.05364	0.03681	0.04809	0.02620
Alanine	0.41603	0.09976	0.42752	0.08584
Asparagine	0.00000	0.00000	0.06239	0.01687
Creatine	0.03060	0.03882	0.02570	0.01856
Creatinine	0.09179	0.03602	0.09227	0.02161
Glutamic acid	0.16056	0.07817	0.10570	0.04209
Glutamine	0.52619	0.10840	0.76153	0.09933
Glycine	0.25530	0.08643	0.29545	0.06167
Histidine	0.06764	0.03145	0.09084	0.01668
Isoleucine	0.04874	0.02264	0.05879	0.01705
Leucine	0.10062	0.03554	0.12440	0.03172
Lysine	0.17885	0.04961	0.24061	0.04254
Methionine	0.06264	0.01778	0.06559	0.02593
N-Dimethylglycine	0.00660	0.02112	0.00630	0.00194
Ornithine	0.06526	0.04145	0.07893	0.03351
Phenylalanine	0.08202	0.03276	0.06313	0.01301
Proline	0.22137	0.09631	0.27262	0.09465
Sarcosine	0.00381	0.00320	0.00496	0.00379
Threonine	0.14205	0.09455	0.19374	0.07430
Tyrosine	0.05319	0.01908	0.05752	0.01280
Valine	0.23508	0.05970	0.26290	0.04941
2-Hydroxybutyric acid	0.08004	0.07177	0.06130	0.08485
Acetic acid	0.02179	0.07550	0.02273	0.02716
Citric acid	0.14567	0.04353	0.16679	0.03569
Formic acid	0.02076	0.01257	0.02198	0.00898
Lactic acid	3.19901	1.43651	3.03921	0.73606
Succinic acid	0.00564	0.00984	0.00493	0.00446
Choline	0.01211	0.00996	0.01900	0.01093
2-Oxoglutaric acid	0.01036	0.00426	0.01268	0.00134
3-Hydroxybutyric acid	0.12403	0.24238	0.09368	0.09362
Acetoacetic acid	0.02390	0.06354	0.01274	0.01571
Acetone	0.05023	0.06652	0.02817	0.02381
Pyruvic acid	0.05775	0.03162	0.05510	0.02984
D-Galactose	0.43680	0.09109	0.00000	0.00000
Glucose	5.09907	2.10745	5.05708	1.12955

Glycerol	0.26460	0.09947	0.24102	0.07529
Dimethylsulfone	0.01774	0.11837	0.01357	0.00614
Fischer's Ratio (calculated)	3.06748	1.22207	3.76908	0.80392
Gln/Glu (calculated)	4.33973	3.71139	8.53654	4.26473

B.I. LISA Parameter (mg/dL)	Mean Value in COVID cohort (n=509)	Standard Deviation in COVID cohort	Mean Value in Healthy Control cohort (n=305)	Standard Deviation in Healthy Control cohort
TPTG	140.73540	74.27033	102.88003	63.21554
TPCH	161.52132	40.35036	211.75233	42.68864
LDCH	83.55028	30.83685	115.89652	31.93444
HDCH	45.87466	11.34474	63.72436	15.51808
TPA1	120.47862	24.08536	160.26702	25.58770
TPA2	25.25487	4.84837	34.81593	5.69773
TPAB	78.82912	21.36134	87.56351	21.92518
LDHD	1.87900	0.72663	1.89033	0.57810
ABA1	0.67328	0.21097	0.55692	0.15352
TBPN	1433.32306	388.40469	1592.13813	398.66301
VLPN	175.90185	71.90502	131.11082	75.98491
IDPN	83.30240	44.00092	89.90062	41.72519
LDPN	1125.46275	349.83974	1339.30407	343.03353
L1PN	206.68809	68.41525	233.61502	75.54545
L2PN	152.90489	60.51144	163.65417	76.94356
L3PN	136.10383	63.16585	174.03937	71.65816
L4PN	139.55580	78.87648	199.14977	84.31521
L5PN	179.23901	86.50847	234.14079	106.93761
L6PN	322.11383	140.89184	349.00243	138.77315
VLTG	90.48570	54.30593	63.38443	48.36920
IDTG	12.96655	12.91329	8.81271	10.60519
LDTG	22.00497	8.41603	18.78830	5.85204
HDTG	10.24322	3.96781	10.35944	4.16623
VLCH	20.87988	11.31667	17.36757	12.21790
IDCH	10.01587	6.71768	11.43023	6.62949
VLFC	10.28517	4.72185	8.02331	4.97229
IDFC	2.74010	1.88719	3.33957	1.99531
LDFC	27.90721	8.63293	34.54577	8.63225
HDFC	12.15398	3.64757	14.74951	4.27482
VLPL	22.24218	10.27850	17.82298	10.98811
IDPL	5.35853	3.88740	6.86062	3.73351
LDPL	50.27369	15.10020	65.68400	15.49057
HDPL	63.59676	14.49552	86.03643	19.48948
HDA1	122.03291	24.86965	160.41616	27.91288
HDA2	26.50703	4.57178	35.00731	5.42419
VLAB	9.67413	3.95452	7.21082	4.17919
IDAB	4.58146	2.42006	4.94423	2.29499

LDAB	61.89770	19.24037	73.65862	18.86610
V1TG	47.27223	35.77080	27.59162	31.12412
V2TG	14.91666	9.58968	10.22613	8.70403
V3TG	12.15637	7.39694	9.18439	7.14776
V4TG	9.29967	4.36956	7.99856	4.54810
V5TG	3.44417	0.82918	2.81767	0.86209
V1CH	8.20707	6.00106	5.24238	5.19599
V2CH	3.37153	2.17087	2.66702	2.22620
V3CH	3.12773	2.22957	3.14034	2.60104
V4CH	4.25506	2.42971	4.76934	3.08815
V5CH	1.43929	0.62111	1.46136	0.71795
V1FC	2.70966	2.42837	2.08923	2.24736
V2FC	1.59287	1.06947	1.05207	0.98825
V3FC	1.64396	1.13601	1.28959	1.18295
V4FC	1.90725	1.18931	2.04622	1.37752
V5FC	0.93939	0.45075	0.63398	0.39799
V1PL	6.84168	5.34513	4.64850	4.95064
V2PL	3.46790	2.19630	2.72672	2.17133
V3PL	3.59014	2.14775	3.27844	2.33834
V4PL	4.44635	2.00368	4.31259	2.34372
V5PL	1.90813	0.65929	1.74309	0.80254
L1TG	7.15735	3.41665	5.43115	2.40849
L2TG	2.57022	1.15277	2.35931	0.85112
L3TG	2.73071	0.87109	2.44774	0.77547
L4TG	2.77751	1.49200	2.48296	1.17134
L5TG	2.77589	1.29904	2.72721	1.39655
L6TG	4.62666	1.73744	3.96613	1.41446
L1CH	18.34994	6.44335	24.43184	8.47669
L2CH	13.63339	6.17850	16.23836	8.72517
L3CH	11.01557	6.19281	16.50507	7.39016
L4CH	11.53627	6.63798	17.35403	7.52652
L5CH	13.04750	6.80342	18.35681	8.57003
L6CH	20.84071	9.18972	23.84384	9.33302
L1FC	5.69211	2.02411	7.52928	2.44276
L2FC	4.92427	1.98473	5.48364	2.69300
L3FC	4.40557	1.79544	5.64521	2.14353
L4FC	3.75035	1.82077	5.32859	1.85767
L5FC	3.99077	1.76138	5.25948	2.00264
L6FC	4.96229	2.20632	6.12577	2.16772
L1PL	11.30988	3.57869	13.91102	4.33453
L2PL	7.93958	3.16546	9.27216	4.40419
L3PL	6.60167	3.18123	9.27663	3.74395
L4PL	6.52222	3.52962	9.68586	3.85215
L5PL	7.36633	3.51402	10.01246	4.31168
L6PL	11.76714	4.58027	13.49325	4.51198
L1AB	11.36727	3.76278	12.84803	4.15480

L2AB	8.40963	3.32803	9.00083	4.23153
L3AB	7.48546	3.47391	9.57162	3.94133
L4AB	7.67514	4.33827	10.95293	4.63726
L5AB	9.85781	4.75774	12.87720	5.88116
L6AB	17.71524	7.74890	19.19426	7.63242
H1TG	3.31759	2.02827	3.40414	2.03684
H2TG	1.85363	0.77541	1.69948	0.82631
H3TG	2.23432	0.76404	2.03237	0.85320
H4TG	3.34695	0.99101	3.39512	1.32674
H1CH	13.87055	7.17739	19.64872	10.26331
H2CH	6.63157	2.13960	9.39505	3.16030
H3CH	7.98866	2.00052	11.43243	2.45340
H4CH	15.43291	4.22437	21.91584	4.78118
H1FC	3.45558	1.81952	5.19410	2.46559
H2FC	1.56194	0.59472	2.30724	0.81278
H3FC	1.62209	0.64480	2.48285	0.74305
H4FC	2.98529	1.11892	4.19872	1.22248
H1PL	16.89525	8.80869	23.42551	12.55148
H2PL	10.76173	3.30578	14.16780	4.63797
H3PL	13.11743	3.09561	17.82662	3.99504
H4PL	21.84574	4.89482	29.77528	5.80918
H1A1	21.90915	12.67968	29.66839	18.02447
H2A1	15.04976	4.14557	20.21485	5.34710
H3A1	22.54780	5.06870	28.90984	5.92074
H4A1	62.21591	13.31761	81.23043	14.60649
H1A2	1.87768	1.27405	2.81568	1.74580
H2A2	2.57941	0.95305	3.64256	1.23501
H3A2	5.08815	1.32109	6.85279	1.56179
H4A2	15.00513	3.83958	20.78751	4.76019

Table S3. Analysis ambulatory COVID-19 vs. Healthy controls

Univariate Analysis (sorted by p-value)

Parameter	FC	log2(FC)	p.adjusted	-log10(p)
Glutamine	0.7083	-0.49758	3.35×10^{-103}	102.47
TPA2	0.74647	-0.42184	5.73×10^{-78}	77.242
Citric acid	0.61261	-0.70695	7.18×10^{-78}	77.144
HDA2	0.77529	-0.36718	2.03×10^{-71}	70.692
TPA1	0.77709	-0.36386	8.45×10^{-66}	65.073
Lysine	0.66423	-0.59024	3.94×10^{-61}	60.404
H3CH	0.7175	-0.47896	3.86×10^{-56}	55.413
HDA1	0.78704	-0.3455	3.86×10^{-56}	55.413
Gln/Glu	0.55374	-0.85272	2.89×10^{-55}	54.539
H4PL	0.75962	-0.39666	1.83×10^{-51}	50.738
HDPL	0.75742	-0.40084	5.53×10^{-51}	50.258
HDCH	0.74054	-0.43336	1.85×10^{-50}	49.732
H3PL	0.75274	-0.40978	2.14×10^{-48}	47.669
H4A1	0.79457	-0.33175	2.39×10^{-44}	43.622
H4CH	0.72985	-0.45433	7.99×10^{-44}	43.097
TPCH	0.78543	-0.34845	1.03×10^{-40}	39.987
H4A2	0.74738	-0.42009	1.72×10^{-40}	39.764
H3A1	0.8017	-0.31887	1.67×10^{-36}	35.778
H2A1	0.76571	-0.38514	9.91×10^{-36}	35.004
H3A2	0.76127	-0.39351	1.56×10^{-34}	33.806
LDCH	0.74659	-0.42161	3.27×10^{-30}	29.486
LDPL	0.78539	-0.34852	4.11×10^{-30}	29.386
V1TG	1.8042	0.85134	1.29×10^{-29}	28.891
H2CH	0.71737	-0.47922	4.28×10^{-29}	28.368
H3FC	0.70039	-0.51377	5.22×10^{-28}	27.282
V5FC	1.5858	0.66517	4.53×10^{-27}	26.344
V1CH	1.6613	0.73228	7.30×10^{-27}	26.137
V2FC	1.5655	0.64666	7.30×10^{-27}	26.137
H2A2	0.73064	-0.45277	1.08×10^{-25}	24.967
Leucine	0.79051	-0.33915	4.21×10^{-25}	24.376
L1FC	0.75508	-0.4053	7.27×10^{-25}	24.139
H2FC	0.69197	-0.53122	3.80×10^{-24}	23.42
V2TG	1.5173	0.60149	9.82×10^{-24}	23.008
TPTG	1.4071	0.49277	2.00×10^{-23}	22.7
L1CH	0.75295	-0.40938	5.92×10^{-23}	22.228
VLTG	1.4812	0.56676	6.18×10^{-23}	22.209
Fischer's Ratio	0.8345	-0.26101	4.65×10^{-22}	21.332
VLPN	1.336	0.41788	4.86×10^{-22}	21.313
VLAB	1.336	0.41788	4.86×10^{-22}	21.313
Dimethylsulfone	1.2427	0.31349	7.31×10^{-22}	21.136

Histidine	0.77163	-0.37401	2.36×10^{-21}	20.627
H2PL	0.76768	-0.38143	3.81×10^{-21}	20.419
V5TG	1.1988	0.26154	7.70×10^{-21}	20.114
H4FC	0.74965	-0.41572	2.73×10^{-20}	19.563
H1FC	0.70387	-0.50661	3.65×10^{-19}	18.438
VLFC	1.3124	0.39219	3.89×10^{-19}	18.41
L4PL	0.68697	-0.54169	4.95×10^{-19}	18.305
LDFC	0.83023	-0.26843	6.60×10^{-18}	17.181
V3TG	1.3623	0.44603	2.96×10^{-17}	16.529
L4CH	0.67625	-0.56438	3.60×10^{-17}	16.444
L1PL	0.80746	-0.30854	1.31×10^{-16}	15.883
V2CH	1.3099	0.38949	1.77×10^{-16}	15.752
V1PL	1.5613	0.64279	1.95×10^{-16}	15.711
VLPL	1.2847	0.36138	4.05×10^{-16}	15.393
Isoleucine	0.83416	-0.2616	4.46×10^{-15}	14.35
L4FC	0.74136	-0.43175	1.22×10^{-14}	13.914
V2PL	1.323	0.40381	2.35×10^{-14}	13.629
H1CH	0.72413	-0.46569	3.03×10^{-14}	13.519
L3CH	0.69609	-0.52266	4.20×10^{-14}	13.377
L5CH	0.7269	-0.46017	7.20×10^{-14}	13.143
L4AB	0.7224	-0.46914	1.52×10^{-13}	12.819
Valine	0.88568	-0.17514	2.08×10^{-13}	12.681
V3FC	1.3114	0.39114	2.82×10^{-13}	12.549
L3PL	0.73805	-0.43821	4.16×10^{-13}	12.381
L4PN	0.72711	-0.45975	4.17×10^{-13}	12.38
LDPN	0.85644	-0.22358	5.04×10^{-13}	12.298
LDAB	0.85644	-0.22358	5.04×10^{-13}	12.298
L1TG	1.2721	0.34724	6.47×10^{-13}	12.189
H1A2	0.69763	-0.51946	8.35×10^{-13}	12.078
Glycine	0.88404	-0.17781	8.73×10^{-13}	12.059
H1PL	0.73888	-0.4366	1.04×10^{-12}	11.983
IDTG	1.6079	0.68515	1.40×10^{-12}	11.855
ABA1	1.1835	0.2431	2.14×10^{-12}	11.671
L5FC	0.77732	-0.36342	5.34×10^{-12}	11.272
VLCH	1.2198	0.28665	1.28×10^{-11}	10.892
HDFC	0.85481	-0.22632	2.27×10^{-11}	10.643
Trimethylamine-N-oxide	0.62666	-0.67424	2.93×10^{-11}	10.533
L5PL	0.75535	-0.40478	5.37×10^{-11}	10.27
Acetic acid	1.0132	0.018976	6.32×10^{-11}	10.199
L6FC	0.83728	-0.25622	3.32×10^{-10}	9.4793
L5PN	0.7814	-0.35586	6.38×10^{-10}	9.1952
L5AB	0.7853	-0.34868	1.38×10^{-9}	8.8601
3-Hydroxybutyric	1.0609	0.085247	3.12×10^{-9}	8.5064

acid				
Ornithine	0.80371	-0.31525	1.06×10^{-8}	7.9755
IDPL	0.81368	-0.29746	1.33×10^{-8}	7.8753
L3FC	0.80979	-0.30438	6.96×10^{-8}	7.1576
L3AB	0.80296	-0.3166	1.27×10^{-7}	6.8968
L3PN	0.80392	-0.31488	1.46×10^{-7}	6.836
L1PN	0.87269	-0.19646	1.98×10^{-7}	6.7039
L1AB	0.87269	-0.19645	1.98×10^{-7}	6.7039
H1A1	0.76713	-0.38246	3.19×10^{-7}	6.4965
V1FC	1.4164	0.5022	4.80×10^{-7}	6.319
V4TG	1.1495	0.20105	5.49×10^{-7}	6.2602
Tyrosine	0.90269	-0.1477	7.43×10^{-7}	6.1291
H3TG	1.1087	0.14888	1.43×10^{-6}	5.8432
L6TG	1.1606	0.21487	2.31×10^{-6}	5.6361
LDTG	1.1456	0.19616	3.02×10^{-6}	5.5206
L6PL	0.89242	-0.16421	4.15×10^{-6}	5.3818
TPAB	0.91263	-0.13189	4.71×10^{-6}	5.3274
TBPN	0.91263	-0.13189	4.71×10^{-6}	5.3274
Formic acid	0.92904	-0.10619	7.05×10^{-6}	5.1516
Succinic acid	1.0564	0.079209	1.85×10^{-5}	4.7319
Acetone	1.4203	0.50621	2.09×10^{-5}	4.6796
Phenylalanine	1.1987	0.26145	2.36×10^{-5}	4.6279
V3PL	1.1233	0.16779	2.40×10^{-5}	4.62
IDFC	0.81849	-0.28896	6.60×10^{-5}	4.1806
L3TG	1.0928	0.128	8.89×10^{-5}	4.0511
L6CH	0.89645	-0.1577	9.67×10^{-5}	4.0148
N-Dimethylglycine	2.3541	1.2352	0.00021631	3.6649
V5PL	1.0774	0.10753	0.0006073	3.2166
H2TG	1.0853	0.11804	0.00070358	3.1527
V3CH	1.0438	0.061787	0.00073097	3.1361
Creatinine	0.95773	-0.062309	0.00084979	3.0707
Acetoacetic acid	1.333	0.41466	0.0017136	2.7661
Glutamic acid	1.2419	0.31252	0.001744	2.7585
IDCH	0.87044	-0.20019	0.001744	2.7585
IDPN	0.90818	-0.13894	0.004568	2.3403
IDAB	0.90821	-0.1389	0.004568	2.3403
Glucose	0.98603	-0.020294	0.0059522	2.2253
L6PN	0.94212	-0.086022	0.013213	1.879
L6AB	0.9427	-0.085126	0.01402	1.8532
L2PL	0.86396	-0.21096	0.015975	1.7966

H4TG	1.0161	0.023041	0.024108	1.6178
L4TG	1.0889	0.12286	0.046022	1.337

Table S4. Analysis COVID-19 vs. Healthy controls

Univariate Analysis (sorted by p-value)

Parameter	FC	log2(FC)	p.adjusted	-log10(p)
Glutamine	0.6841	-0.54772	1.04×10^{-111}	110.98
TPA2	0.72538	-0.46319	2.44×10^{-94}	93.613
HDA2	0.75719	-0.40128	1.02×10^{-87}	86.991
TPA1	0.75174	-0.4117	1.27×10^{-83}	82.896
Citric acid	0.62126	-0.68673	9.62×10^{-79}	78.017
Lysine	0.65143	-0.61831	6.40×10^{-76}	75.194
HDA1	0.76073	-0.39455	2.91×10^{-73}	72.536
H3CH	0.69877	-0.51711	1.27×10^{-70}	69.896
Gln/Glu	0.535	-0.9024	5.59×10^{-67}	66.252
HDPL	0.73918	-0.43599	2.20×10^{-66}	65.658
HDCH	0.71989	-0.47415	9.75×10^{-65}	64.011
H4PL	0.73369	-0.44676	3.58×10^{-64}	63.446
H3PL	0.73583	-0.44255	1.41×10^{-61}	60.852
H4A1	0.76592	-0.38474	1.20×10^{-57}	56.92
H4A2	0.72183	-0.47026	1.35×10^{-51}	50.87
TPCH	0.76278	-0.39065	6.19×10^{-51}	50.208
H4CH	0.70392	-0.50651	6.37×10^{-51}	50.196
H3A1	0.77994	-0.35857	5.91×10^{-49}	48.228
H2A1	0.74449	-0.42567	1.74×10^{-48}	47.761
H3A2	0.74249	-0.42955	1.18×10^{-45}	44.929
H3FC	0.65433	-0.61191	1.80×10^{-37}	36.745
H2CH	0.70586	-0.50255	9.34×10^{-37}	36.03
LDPL	0.76539	-0.38574	2.83×10^{-36}	35.549
H2A2	0.70813	-0.49791	2.73×10^{-34}	33.564
LDCH	0.72038	-0.47318	1.50×10^{-33}	32.823
V5FC	1.5719	0.65249	2.56^{-33}	32.592
Fischer's Ratio	0.80383	-0.31503	1.37×10^{-32}	31.864
V1TG	1.716	0.77907	2.38×10^{-32}	31.624
V2FC	1.5272	0.6109	5.64×10^{-31}	30.249
H2FC	0.67684	-0.56312	1.08×10^{-29}	28.967
V1CH	1.5871	0.6664	2.31×10^{-28}	27.636
VLPN	1.3416	0.42398	7.84×10^{-28}	27.106
VLAB	1.3416	0.42397	7.84×10^{-28}	27.106
V5TG	1.2223	0.28965	2.24×10^{-27}	26.651
L1FC	0.75605	-0.40345	2.41×10^{-27}	26.617
H1FC	0.66562	-0.58722	2.90×10^{-27}	26.537
H4FC	0.71136	-0.49135	1.15×10^{-26}	25.939
H2PL	0.75959	-0.39671	2.23×10^{-26}	25.652
L1CH	0.75049	-0.41409	2.65×10^{-26}	25.577
L4PL	0.65059	-0.62017	2.11×10^{-25}	24.675
V2TG	1.4587	0.54466	4.29×10^{-25}	24.367

Histidine	0.74462	-0.42542	7.02×10^{-25}	24.153
Leucine	0.80476	-0.31336	1.72×10^{-24}	23.764
TPTG	1.368	0.45202	6.86×10^{-24}	23.164
VLTG	1.4276	0.51356	8.99×10^{-24}	23.046
LDFC	0.80783	-0.30787	3.19×10^{-23}	22.497
Dimethylsulfone	1.3473	0.43009	2.45×10^{-22}	21.61
L4CH	0.64172	-0.63999	7.34×10^{-22}	21.134
VLFC	1.2819	0.3583	2.37×10^{-20}	19.626
L4FC	0.70304	-0.50833	3.09×10^{-20}	19.51
L1TG	1.3178	0.39817	1.30×10^{-18}	17.885
H1CH	0.70565	-0.50298	1.60×10^{-18}	17.795
L4AB	0.68527	-0.54526	1.69×10^{-18}	17.773
L3CH	0.66506	-0.58845	3.27×10^{-18}	17.486
HDFC	0.82447	-0.27846	3.83×10^{-18}	17.417
V3TG	1.3283	0.40955	3.85×10^{-18}	17.415
L1PL	0.81302	-0.29864	7.48×10^{-18}	17.126
L5CH	0.70177	-0.51092	9.08×10^{-18}	17.042
L3PL	0.71021	-0.49368	1.68×10^{-17}	16.775
V1PL	1.4874	0.57278	3.03×10^{-17}	16.518
L4PN	0.69663	-0.52153	3.05×10^{-17}	16.515
Glycine	0.8641	-0.21074	4.18×10^{-17}	16.378
H1A2	0.66862	-0.58074	8.71×10^{-17}	16.06
Isoleucine	0.82895	-0.27064	1.06×10^{-16}	15.977
VLPL	1.2479	0.31956	1.36×10^{-16}	15.867
LDPN	0.84033	-0.25097	1.44×10^{-16}	15.842
LDAB	0.84033	-0.25097	1.44×10^{-16}	15.842
H1PL	0.72123	-0.47146	2.19×10^{-16}	15.66
V2CH	1.2642	0.33818	5.35×10^{-16}	15.271
ABA1	1.2089	0.27374	5.38×10^{-16}	15.269
V3FC	1.292	0.36957	1.63×10^{-15}	14.788
L5FC	0.7559	-0.40372	2.93×10^{-15}	14.533
L5PL	0.72952	-0.45499	2.12×10^{-14}	13.673
V2PL	1.2718	0.34689	3.45×10^{-14}	13.462
Valine	0.89418	-0.16137	6.55×10^{-13}	12.184
VLCH	1.2022	0.26572	7.00×10^{-13}	12.155
L5PN	0.76034	-0.39529	7.55×10^{-13}	12.122
Phenylalanine	1.2936	0.37136	1.94×10^{-12}	11.712
L6FC	0.81138	-0.30155	1.94×10^{-12}	11.712
IDPL	0.7708	-0.37557	2.83×10^{-12}	11.548
L5AB	0.76712	-0.38248	2.90×10^{-12}	11.538
Acetic acid	0.95991	-0.05903	8.01×10^{-12}	11.096
Trimethylamine-N-oxide	0.64414	-0.63456	1.22×10^{-11}	10.915
L3FC	0.78183	-0.35508	1.22×10^{-11}	10.915
IDTG	1.507	0.59166	5.21×10^{-11}	10.283

L3AB	0.78125	-0.35614	2.14×10^{-10}	9.6696
Acetone	1.7595	0.8152	2.18×10^{-10}	9.6625
L3PN	0.78201	-0.35474	2.45×10^{-10}	9.6106
H1A1	0.73887	-0.4366	2.78×10^{-10}	9.5567
Ornithine	0.79665	-0.32799	2.98×10^{-10}	9.5254
V4TG	1.1627	0.21744	4.29×10^{-09}	8.3672
LDTG	1.1712	0.228	5.18×10^{-09}	8.2858
TPAB	0.90025	-0.1516	3.12×10^{-08}	7.5063
TBPN	0.90025	-0.1516	3.12×10^{-08}	7.5063
L6PL	0.87311	-0.19577	3.39×10^{-08}	7.47
L6TG	1.1665	0.22224	4.25×10^{-08}	7.3718
L3TG	1.1156	0.15783	4.02×10^{-07}	6.3954
L1PN	0.88474	-0.17668	5.55×10^{-07}	6.2554
L1AB	0.88475	-0.17666	5.55×10^{-07}	6.2554
L6CH	0.87356	-0.19502	9.26×10^{-07}	6.0335
H3TG	1.1	0.13751	2.49×10^{-06}	5.603
Tyrosine	0.91967	-0.12082	1.02×10^{-05}	4.9903
V5PL	1.0958	0.13193	1.16×10^{-05}	4.9365
V3PL	1.1021	0.14024	1.98×10^{-05}	4.7028
IDFC	0.81631	-0.29282	2.31×10^{-05}	4.6362
V1FC	1.3128	0.3926	2.74×10^{-05}	4.5615
Formic acid	0.95039	-0.073414	5.96×10^{-05}	4.2247
N-Dimethylglycine	2.3684	1.2439	6.47×10^{-05}	4.1893
Glutamic acid	1.2456	0.31688	0.00022707	3.6438
H2TG	1.0907	0.12527	0.00024591	3.6092
V3CH	1.0264	0.037585	0.00075492	3.1221
3-Hydroxybutyric acid	1.3828	0.46758	0.00078475	3.1053
L6PN	0.92038	-0.1197	0.0010173	2.9925
L6AB	0.92249	-0.11639	0.0014059	2.8521
IDCH	0.87393	-0.19442	0.0019649	2.7067
L2PL	0.85801	-0.22093	0.0038782	2.4114
V4PL	1.031	0.044066	0.007102	2.1486
L4TG	1.1144	0.1563	0.010861	1.9641
L2TG	1.0901	0.12452	0.01209	1.9176
IDPN	0.92538	-0.11187	0.017608	1.7543
IDAB	0.92563	-0.11149	0.018221	1.7394
L2CH	0.84157	-0.24884	0.02025	1.6936
Alanine	0.97312	-0.039304	0.026962	1.5692
Succinic acid	1.2426	0.31342	0.030993	1.5087

Table S5. Analysis of hospitalized sub-cohort vs. outpatient COVID-19 cohort

Univariate analysis (sorted by p-value)

**Parameter**	**FC**	**log2(FC)**	**p.adjusted**	**-log10(p)**
Iron	0.53218	-0.91002	2.83×10^{-16}	15.548
Ferritin	2.3278	1.219	1.00×10^{-14}	13.999
LDH	1.284	0.36064	7.30×10^{-14}	13.136
Glyc/SPC	1.3147	0.39473	3.98×10^{-13}	12.4
Transferrin	0.83146	-0.26628	1.33×10^{-12}	11.877
GlycA	1.1505	0.20232	4.62×10^{-12}	11.335
Acetoacetic acid	2.4373	1.2853	5.00×10^{-12}	11.301
HDA1	0.8681	-0.20407	5.00×10^{-12}	11.301
Glyc	1.1493	0.2007	5.00×10^{-12}	11.301
CRP	2.9003	1.5362	7.85×10^{-12}	11.105
TPA1	0.87129	-0.19877	7.85×10^{-12}	11.105
GlycB	1.146	0.19665	1.41×10^{-11}	10.851
H3FC	0.74193	-0.43064	1.69×10^{-11}	10.773
H4A1	0.85772	-0.22142	3.21×10^{-11}	10.494
Phenylalanine	1.3087	0.38819	1.75×10^{-10}	9.7577
3-Hydroxybutyric acid	2.2823	1.1905	7.38×10^{-10}	9.1317
Creatinine	1.2796	0.3557	4.70×10^{-9}	8.3284
TPA2	0.88853	-0.1705	4.70×10^{-9}	8.3284
H4PL	0.86532	-0.2087	1.29×10^{-8}	7.8909
Glutamine	0.87548	-0.19186	2.01×10^{-8}	7.696
GFR (CKD-EPI)	0.85087	-0.23299	5.98×10^{-8}	7.2232
Acetone	1.9308	0.94923	8.46×10^{-8}	7.0726
HDA2	0.90784	-0.13949	1.27×10^{-7}	6.8976
SPC	0.84603	-0.24121	4.63×10^{-7}	6.3346
H1FC	0.78659	-0.34632	1.58×10^{-6}	5.8027
Succinic acid	1.6827	0.75078	1.72×10^{-6}	5.7639
H4FC	0.79888	-0.32394	1.72×10^{-6}	5.7639
TPCH	0.88624	-0.17422	1.72×10^{-6}	5.7639
H4A2	0.86514	-0.20899	2.98×10^{-6}	5.5251
H3A1	0.89289	-0.16344	2.98×10^{-6}	5.5251
Transferrin saturation	0.62377	-0.68091	3.45×10^{-6}	5.4618
L4CH	0.75295	-0.40936	3.77×10^{-6}	5.4238
HDCH	0.89001	-0.16811	3.85×10^{-6}	5.4145
AST	1.2844	0.3611	4.28×10^{-6}	5.3688
H4CH	0.85956	-0.21832	5.74×10^{-6}	5.2409
LDCH	0.8614	-0.21525	5.74×10^{-6}	5.2409
Age	1.131	0.17765	6.98×10^{-6}	5.1563

HDFC	0.86032	-0.21706	7.11×10^{-6}	5.1479
L4FC	0.80059	-0.32087	7.34×10^{-6}	5.1346
Lymphocytes (abs.)	0.83053	-0.2679	7.34×10^{-6}	5.1346
HDPL	0.90501	-0.144	2.50×10^{-5}	4.602
L4PL	0.7804	-0.35772	2.88×10^{-5}	4.5408
LDFC	0.89358	-0.16233	5.00×10^{-5}	4.3007
Urea	1.2914	0.36888	5.47×10^{-5}	4.2622
Fischer's Ratio	0.84984	-0.23473	7.12×10^{-5}	4.1474
H4TG	0.89081	-0.16681	9.29×10^{-5}	4.032
L3CH	0.82443	-0.27853	0.00010502	3.9787
H2A1	0.89068	-0.16703	0.00010502	3.9787
Creatinine	1.1573	0.21078	0.00011635	3.9342
LDPL	0.89952	-0.15277	0.00011635	3.9342
Methionine	0.8915	-0.1657	0.00013082	3.8833
H3CH	0.89704	-0.15676	0.00029356	3.5323
H3PL	0.91139	-0.13387	0.00033329	3.4772
L3FC	0.86566	-0.20812	0.00040166	3.3961
L4AB	0.81901	-0.28805	0.00062065	3.2072
L3PL	0.85096	-0.23284	0.00062065	3.2072
L4PN	0.81998	-0.28634	0.00063065	3.2002
d-dimers	1.2072	0.27162	0.00093836	3.0276
V5CH	1.1553	0.20825	0.0013677	2.864
L1TG	1.1418	0.19127	0.0017436	2.7586
GGT	1.1924	0.25385	0.0018508	2.7326
V1FC	0.71176	-0.49054	0.0020636	2.6854
L5CH	0.85632	-0.22378	0.0022207	2.6535
V2CH	0.86213	-0.21402	0.0026743	2.5728
H2A2	0.87845	-0.18696	0.0026743	2.5728
L5PL	0.86494	-0.20933	0.0037202	2.4294
L6FC	0.87543	-0.19194	0.0038218	2.4177
L2TG	1.1282	0.17406	0.0042013	2.3766
H3A2	0.90267	-0.14773	0.0044413	2.3525
LDAB	0.92581	-0.11122	0.0045652	2.3405
LDPN	0.92581	-0.11121	0.0045652	2.3405
Platelets	0.94113	-0.087535	0.0055482	2.2558
IDTG	0.75291	-0.40944	0.0056291	2.2496
Glycine	0.91098	-0.13451	0.007602	2.1191
L5PN	0.89361	-0.16228	0.0078343	2.106
V5TG	1.0776	0.10786	0.0078343	2.106
L3PN	0.89239	-0.16426	0.0081263	2.0901
L3AB	0.89273	-0.1637	0.0081263	2.0901
V1CH	0.82285	-0.2813	0.010235	1.9899
V2PL	0.84736	-0.23895	0.010235	1.9899
L5AB	0.89945	-0.15289	0.01029	1.9876

L5FC	0.88691	-0.17314	0.011969	1.9219
Histidine	0.86188	-0.21444	0.012758	1.8942
IDPL	0.7939	-0.33298	0.013581	1.8671
V2TG	0.84761	-0.23853	0.01436	1.8428
L3TG	1.0824	0.11424	0.014748	1.8313
LDTG	1.088	0.12171	0.024612	1.6088
VLTG	0.85715	-0.22237	0.025665	1.5907
VLPL	0.88727	-0.17255	0.025665	1.5907
V4FC	1.1237	0.16829	0.025665	1.5907
L6CH	0.89757	-0.15591	0.025665	1.5907
TPTG	0.89011	-0.16794	0.028493	1.5453
V4CH	1.1101	0.15071	0.028921	1.5388
TPAB	0.94647	-0.079377	0.029343	1.5325
TBPN	0.94647	-0.079375	0.029343	1.5325
Creatine	0.95426	-0.067545	0.031012	1.5085
Gln/Glu	0.85068	-0.23332	0.033608	1.4736
ABA1	1.0847	0.1173	0.034178	1.4663
V1TG	0.80567	-0.31174	0.035061	1.4552
V4PL	1.0818	0.11342	0.038397	1.4157
Alanine	0.94543	-0.080956	0.038397	1.4157
L6PL	0.91321	-0.13098	0.038861	1.4105
Leucine	1.071	0.098962	0.038861	1.4105
H2FC	0.91371	-0.1302	0.040096	1.3969
Glucose	1.0891	0.12317	0.042314	1.3735
V3TG	0.90148	-0.14963	0.044147	1.3551
Neutrophils (absolut)	1.1513	0.20329	0.048274	1.3163
VLFC	0.90838	-0.13863	0.048274	1.3163

Table S6. Analysis of hospitalized male sub-cohort vs. male outpatient sub-cohort

Univariate analysis (sorted by p-value)

Parameter	FC	log2(FC)	p.adjusted	-log10(p)
Iron	0.47661	-1.0691	1.89×10^{-14}	13.724
Transferrin saturation	0.54894	-0.86528	2.49×10^{-11}	10.605
H3FC	0.7004	-0.51375	5.85×10^{-8}	7.2331
TPA1	0.87159	-0.19828	4.94×10^{-7}	6.306
HDA1	0.87314	-0.19571	4.94×10^{-7}	6.306
H4A1	0.83446	-0.26109	5.30×10^{-7}	6.2759
TPA2	0.8627	-0.21306	6.32×10^{-7}	6.1995
Glyc/SPC	1.2704	0.34526	6.49×10^{-7}	6.188
LDH	1.2465	0.31793	1.18×10^{-6}	5.9272
CRP	2.5243	1.3359	1.42×10^{-6}	5.8483
H4PL	0.83685	-0.25696	3.63×10^{-6}	5.4405
HDA2	0.88452	-0.17703	4.22×10^{-6}	5.3751
Acetoacetic acid	2.3403	1.2267	4.42×10^{-6}	5.3544
Transferrin	0.86658	-0.2066	4.42×10^{-6}	5.3544
3-Hydroxybutyric acid	2.3135	1.2101	9.80×10^{-6}	5.0089
GlycA	1.1231	0.16744	1.03×10^{-5}	4.9872
Phenylalanine	1.2538	0.32628	1.06×10^{-5}	4.9739
Glyc	1.1214	0.16532	1.22×10^{-5}	4.9152
Fischer's Ratio	0.80288	-0.31675	1.78×10^{-5}	4.7488
Acetone	2.0356	1.0255	1.90×10^{-5}	4.7203
H4A2	0.82384	-0.27956	1.90×10^{-5}	4.7203
Glutamine	0.86871	-0.20305	1.90×10^{-5}	4.7203
TPCH	0.84475	-0.2434	2.38×10^{-5}	4.624
GlycB	1.1174	0.16019	2.82×10^{-5}	4.5491
V2CH	0.74107	-0.43231	3.78×10^{-5}	4.4224
V2PL	0.72016	-0.47361	8.23×10^{-5}	4.0844
Succinic acid	1.7932	0.84257	8.73×10^{-5}	4.0588
Choline	0.70887	-0.49641	0.00014506	3.8385
H3A2	0.87046	-0.20014	0.00017333	3.7611
SPC	0.84732	-0.23903	0.00019122	3.7185
VLTG	0.7532	-0.4089	0.0002163	3.6649
V2TG	0.7178	-0.47834	0.00024299	3.6144
H3A1	0.88723	-0.17263	0.00026062	3.584
Methionine	0.86478	-0.20959	0.00026245	3.5809
VLPL	0.80279	-0.3169	0.00036421	3.4387
LDCH	0.80788	-0.30779	0.00036421	3.4387
IDTG	0.62263	-0.68355	0.00037575	3.4251
LDFC	0.85167	-0.23164	0.00037891	3.4215
V1FC	0.59779	-0.74229	0.00040253	3.3952

TPTG	0.80151	-0.3192	0.00045423	3.3427
H4FC	0.78441	-0.35032	0.00054706	3.262
L4CH	0.66407	-0.59058	0.0006243	3.2046
H4CH	0.83852	-0.25408	0.0006243	3.2046
LDPL	0.85408	-0.22756	0.00063344	3.1983
L4PL	0.70385	-0.50666	0.00069916	3.1554
IDPL	0.65057	-0.62023	0.00071154	3.1478
L5CH	0.75912	-0.39759	0.00073281	3.135
H1FC	0.82253	-0.28187	0.00079156	3.1015
V1CH	0.74331	-0.42797	0.00090733	3.0422
V3TG	0.78496	-0.3493	0.0010363	2.9845
V1TG	0.71157	-0.49092	0.0010699	2.9707
V5CH	1.2438	0.31474	0.0012051	2.919
L4FC	0.7425	-0.42954	0.0012669	2.8973
V2FC	0.78738	-0.34486	0.001313	2.8817
H4TG	0.86526	-0.20879	0.001313	2.8817
H3CH	0.89425	-0.16126	0.001313	2.8817
VLFC	0.83547	-0.25933	0.0013319	2.8755
HDFC	0.88067	-0.18332	0.0013319	2.8755
V1PL	0.70604	-0.50218	0.0017578	2.755
Ferritin	1.655	0.7268	0.0017876	2.7477
V3PL	0.80126	-0.31965	0.0020752	2.6829
L6FC	0.78222	-0.35436	0.002883	2.5402
L5PL	0.77278	-0.37188	0.0030174	2.5204
Lymphocytes (abs.)	0.86898	-0.20261	0.0040841	2.3889
H3PL	0.90653	-0.14157	0.0041075	2.3864
L4AB	0.72314	-0.46766	0.0044704	2.3497
L5PN	0.79456	-0.33177	0.0044704	2.3497
HDCH	0.91398	-0.12976	0.0044704	2.3497
LDAB	0.87011	-0.20074	0.0047904	2.3196
LDPN	0.87011	-0.20073	0.0047904	2.3196
L6CH	0.79195	-0.33652	0.0050396	2.2976
HDPL	0.92393	-0.11414	0.0056642	2.2469
L5AB	0.8122	-0.30009	0.0063546	2.1969
Platelets	0.90552	-0.14317	0.0072406	2.1402
GFR (CKD-EPI)	0.88488	-0.17644	0.0076917	2.114
H2A2	0.84922	-0.23579	0.0088137	2.0548
VLCH	0.85696	-0.22269	0.0088137	2.0548
TPAB	0.89203	-0.16484	0.0093797	2.0278
TBPN	0.89203	-0.16484	0.0093797	2.0278
L5FC	0.81088	-0.30243	0.010311	1.9867
Alanine	0.91943	-0.12119	0.010679	1.9715
Creatinine	1.1912	0.25242	0.012104	1.9171
L4PN	0.75262	-0.41001	0.012282	1.9107

Lysine	0.87033	-0.20037	0.013844	1.8588
H2A1	0.90824	-0.13885	0.01486	1.828
L6PL	0.81876	-0.28849	0.017248	1.7633
d-dimers	1.0524	0.073692	0.017248	1.7633
Age	1.0824	0.11427	0.02043	1.6897
L3FC	0.85446	-0.22691	0.021058	1.6766
V3CH	0.82679	-0.27441	0.023217	1.6342
Hb	0.96334	-0.053884	0.025924	1.5863
L1TG	1.128	0.17378	0.027044	1.5679
L3CH	0.78374	-0.35156	0.031586	1.5005
LDHD	0.89461	-0.16066	0.033931	1.4694
V3FC	0.84091	-0.24998	0.034292	1.4648
V5TG	1.0853	0.1181	0.035238	1.453
L2TG	1.1124	0.15369	0.036006	1.4436
Isoleucine	0.87483	-0.19292	0.042009	1.3767
L6PN	0.809	-0.30579	0.048835	1.3113
L6AB	0.80982	-0.30433	0.049764	1.3031
L3PL	0.83557	-0.25916	0.049764	1.3031
AST	1.1705	0.22711	0.049764	1.3031

Analysis of hospitalized male sub-cohort >60 yrs. vs. outpatient male sub-cohort > 60 yrs. (n=45 vs. 73)

**Parameter**	**FC**	**log2(FC)**	**p.adjusted**	**-log10(p)**
TPA1	0.82588	-0.27599	2.62×10^{-5}	4.581
Iron	0.53888	-0.89198	5.33×10^{-5}	4.2735
HDA1	0.83125	-0.26665	5.51×10^{-5}	4.2588
H3FC	0.66568	-0.5871	9.44×10^{-5}	4.0249
TPA2	0.83985	-0.2518	9.44×10^{-5}	4.0249
H4A1	0.82965	-0.26943	0.00032782	3.4844
HDA2	0.86588	-0.20776	0.00058453	3.2332
H3A1	0.84325	-0.24596	0.00058985	3.2293
Transferrin saturation	0.61823	-0.6938	0.00072768	3.1381
TPCH	0.81164	-0.30108	0.00072768	3.1381
Glyc/SPC	1.2856	0.36242	0.00091135	3.0403
H1FC	0.70872	-0.49671	0.00096334	3.0162
HDFC	0.80869	-0.30635	0.0010806	2.9663
H4PL	0.84733	-0.239	0.0015395	2.8126
V2CH	0.6647	-0.58923	0.0016427	2.7844
H3A2	0.83946	-0.25247	0.001812	2.7418
H3CH	0.8409	-0.25	0.0021206	2.6735
Transferrin	0.86103	-0.21586	0.0023184	2.6348
SPC	0.80752	-0.30843	0.0028408	2.5466
IDTG	0.49189	-1.0236	0.0034394	2.4635

H4FC	0.74723	-0.42038	0.003951	2.4033
HDPL	0.87297	-0.196	0.003951	2.4033
TPTG	0.72972	-0.45458	0.0039625	2.402
CRP	2.2051	1.1408	0.0047541	2.3229
VLTG	0.67997	-0.55646	0.0050562	2.2962
IDPL	0.56008	-0.8363	0.0053983	2.2677
H2A1	0.85604	-0.22424	0.0058384	2.2337
HDCH	0.85018	-0.23417	0.0063518	2.1971
H4A2	0.82946	-0.26975	0.0076467	2.1165
H4CH	0.83175	-0.26579	0.0085169	2.0697
Glutamine	0.87114	-0.19903	0.0085169	2.0697
Choline	0.70991	-0.49429	0.008574	2.0668
L6CH	0.75024	-0.41457	0.008574	2.0668
V1CH	0.62539	-0.67717	0.0089181	2.0497
V1FC	0.49273	-1.0211	0.0091666	2.0378
V2PL	0.6673	-0.58359	0.0091666	2.0378
H3PL	0.87785	-0.18795	0.010839	1.965
Age	1.0529	0.074308	0.010901	1.9625
LDCH	0.80746	-0.30853	0.011524	1.9384
VLPL	0.75754	-0.4006	0.013125	1.8819
H2A2	0.79123	-0.33783	0.013125	1.8819
3-Hydroxybutyric acid	2.1588	1.1103	0.013157	1.8808
VLFC	0.7744	-0.36885	0.013157	1.8808
L5CH	0.74324	-0.42809	0.013672	1.8642
L6FC	0.75367	-0.408	0.014396	1.8418
LDH	1.1498	0.20133	0.016879	1.7727
V2TG	0.65704	-0.60594	0.017213	1.7642
Succinic acid	1.7219	0.78404	0.019165	1.7175
V2FC	0.7063	-0.50165	0.020982	1.6782
L6PN	0.76045	-0.39508	0.020982	1.6782
Hb	0.95061	-0.073073	0.024488	1.611
LDFC	0.85958	-0.2183	0.025125	1.5999
L6AB	0.769	-0.37894	0.025167	1.5992
V1PL	0.61795	-0.69444	0.027127	1.5666
V1TG	0.61861	-0.69289	0.028773	1.541
V3TG	0.72644	-0.46109	0.029051	1.5368
Acetoacetic acid	2.0085	1.0061	0.029924	1.524
GlycA	1.0874	0.12094	0.029924	1.524
L4PL	0.74148	-0.43152	0.031974	1.4952
VLCH	0.78586	-0.34765	0.031974	1.4952
Formic acid	1.2363	0.30602	0.031974	1.4952
d-dimers	1.1802	0.23908	0.031974	1.4952
Phenylalanine	1.1676	0.22356	0.031974	1.4952
Glyc	1.0862	0.11923	0.031974	1.4952

LDPL	0.8677	-0.20474	0.032324	1.4905
V5CH	1.2412	0.3117	0.032549	1.4875
AST	1.2813	0.35757	0.03373	1.472
Fischer's Ratio	0.87403	-0.19425	0.036445	1.4384
Methionine	0.87683	-0.18962	0.040822	1.3891
GlycB	1.0835	0.11564	0.040822	1.3891
BMI	0.93116	-0.1029	0.045837	1.3388

Analysis of hospitalized male sub-cohort <60 yrs. vs. outpatient male sub-cohort <60 yrs. (n=42 vs. 92)

**Parameter**	**FC**	**log2(FC)**	**p.ajusted**	**-log10(p)**
Iron	0.42809	-1.224	3.56×10^{-9}	8.4486
Transferrin saturation	0.49404	-1.0173	7.56×10^{-8}	7.1216
Acetoacetic acid	2.7184	1.4427	4.87×10^{-5}	4.3128
LDH	1.3462	0.42887	5.38×10^{-5}	4.2693
Acetone	2.1378	1.0961	0.000103	3.9872
Phenylalanine	1.3499	0.43285	0.00045778	3.3393
GlycA	1.1559	0.20903	0.00060881	3.2155
Glyc	1.1537	0.20625	0.00065485	3.1839
CRP	2.8692	1.5206	0.0010356	2.9848
3-Hydroxybutyric acid	2.3982	1.2619	0.0010356	2.9848
GlycB	1.1478	0.19889	0.0010356	2.9848
Glyc/SPC	1.2665	0.34084	0.0013403	2.8728
H3FC	0.73477	-0.44463	0.001435	2.8431
Fischer's Ratio	0.75979	-0.39634	0.001435	2.8431
Platelets	0.76842	-0.38003	0.0014639	2.8345
H4PL	0.82444	-0.27851	0.0020076	2.6973
H4A1	0.83956	-0.25229	0.0023446	2.6299
Ferritin	2.3199	1.214	0.0028661	2.5427
Glutamine	0.86709	-0.20575	0.0045469	2.3423
H4A2	0.82286	-0.28128	0.005564	2.2546
Transferrin	0.87682	-0.18965	0.0061194	2.2133
Succinic acid	1.8174	0.86184	0.0067054	2.1736
TPA2	0.8872	-0.17267	0.011586	1.9361
Methionine	0.8587	-0.21978	0.012628	1.8986
L5PL	0.77636	-0.3652	0.013059	1.8841
HDA1	0.91269	-0.1318	0.013059	1.8841
L4CH	0.67504	-0.56696	0.017312	1.7617
V2PL	0.77773	-0.36266	0.017622	1.754
Lymphocytes (abs.)	0.73689	-0.44048	0.017644	1.7534

HDA2	0.90493	-0.14413	0.017644	1.7534
L4FC	0.72821	-0.45757	0.023346	1.6318
L4PL	0.68115	-0.55395	0.023401	1.6308
V2TG	0.78267	-0.35352	0.023401	1.6308
TPA1	0.91511	-0.12798	0.023401	1.6308
LDFC	0.85072	-0.23324	0.025696	1.5901
H4TG	0.86428	-0.21043	0.026679	1.5738
LDPL	0.84723	-0.23917	0.028034	1.5523
LDCH	0.8168	-0.29195	0.041594	1.381
V2CH	0.82348	-0.28019	0.041594	1.381
L5PN	0.81454	-0.29594	0.045927	1.3379
L5FC	0.83502	-0.26012	0.045927	1.3379
VLPL	0.85303	-0.22932	0.045927	1.3379
H4CH	0.84543	-0.24224	0.047257	1.3255
V5CH	1.2386	0.30867	0.049759	1.3031

Table S7. Analysis of hospitalized female sub-cohort vs. female outpatient sub-cohort

Univariate Analysis (sorted by p-value)

**Parameter**	**FC**	**log2(FC)**	**p.ajusted**	**-log10(p)**
Ferritin	2.9936	1.5819	5.27×10^{-9}	8.2782
LDH	1.3056	0.38473	1.19×10^{-5}	4.9236
GlycA	1.1785	0.2369	1.99×10^{-5}	4.7012
Glyc	1.1772	0.23538	1.99×10^{-5}	4.7012
GlycB	1.1734	0.23071	2.58×10^{-5}	4.5886
Transferrin	0.80966	-0.30462	2.61×10^{-5}	4.5837
GFR (CKD-EPI)	0.84389	-0.24488	3.56×10^{-5}	4.4487
Iron	0.61572	-0.69965	6.66×10^{-5}	4.1767
Acetoacetic acid	1.9185	0.93995	0.00011696	3.932
CRP	3.3151	1.7291	0.00036264	3.4405
AST	1.3357	0.41764	0.0015162	2.8193
Creatinine	1.1391	0.18787	0.0017226	2.7638
Glyc/SPC	1.2349	0.30444	0.001739	2.7597
Phenylalanine	1.3278	0.40906	0.0018553	2.7316
Age	1.1763	0.2343	0.0036294	2.4402
Lymphocytes (abs.)	0.77328	-0.37095	0.0073894	2.1314
GGT	1.5579	0.63963	0.0080641	2.0934
3-Hydroxybutyric acid	1.6987	0.76442	0.0093781	2.0279
L2TG	1.2218	0.289	0.018651	1.7293
H4A1	0.91243	-0.13222	0.018651	1.7293
L1TG	1.2122	0.27759	0.021149	1.6747

Glutamine	0.90575	-0.14282	0.035565	1.449
HDA1	0.91773	-0.12386	0.035565	1.449
LDTG	1.1733	0.23061	0.036047	1.4431
L3TG	1.1587	0.21248	0.037287	1.4284
L1AB	1.1472	0.19815	0.037287	1.4284
L1PN	1.1471	0.19804	0.037287	1.4284
TPA1	0.92251	-0.11636	0.042051	1.3762

Analysis of hospitalized female sub-cohort >60 yrs. vs. outpatient female sub-cohort >60 yrs. (n=25 vs. 61)

**Parameter**	**FC**	**log2(FC)**	**p.ajusted**	**-log10(p)**
Iron	0.44144	-1.1797	2.53×10^{-05}	4.5968
Transferrin saturation	0.5311	-0.91294	0.00033696	3.4724
Ferritin	1.9705	0.97853	0.0033791	2.4712
GlycA	1.1498	0.2014	0.0033791	2.4712
Glyc	1.1454	0.19589	0.0037426	2.4268
Glyc/SPC	1.248	0.31959	0.0097161	2.0125
LDH	1.2179	0.28438	0.0097161	2.0125
GlycB	1.1327	0.17978	0.0097161	2.0125
L1AB	1.2261	0.29413	0.043552	1.361
L1PN	1.2259	0.29389	0.043552	1.361
L1PL	1.2093	0.27416	0.043552	1.361
Transferrin	0.83038	-0.26815	0.045351	1.3434

Analysis of hospitalized female sub-cohort <60 yrs. vs. outpatient female sub-cohort <60 yrs. (n=17 v 148)

**Parameter**	**FC**	**log2(FC)**	**p.ajusted**	**-log10(p)**
Ferritin	3.7796	1.9182	0.0012914	2.8889
L3CH	0.76245	-0.39128	0.0083442	2.0786
AST	1.634	0.70844	0.028548	1.5444
GFR (CKD-EPI)	0.88588	-0.17481	0.028548	1.5444
H4FC	0.83311	-0.26342	0.0373	1.4283
Creatinine	1.1456	0.19607	0.0373	1.4283
Acetoacetic acid	1.8903	0.91862	0.039275	1.4059
Neutrophils (absolut)	1.6072	0.68459	0.043808	1.3584
LDH	1.3175	0.39782	0.043808	1.3584
L4FC	0.81043	-0.30324	0.043808	1.3584
L3PN	0.82022	-0.28593	0.043808	1.3584
L3AB	0.82361	-0.27997	0.043808	1.3584

L3PL	0.79664	-0.328	0.044118	1.3554
LDCH	0.84403	-0.24463	0.047742	1.3211
GlycB	1.1732	0.23045	0.047742	1.3211

Table S8. Hospitalization Biomarker Analysis

Parameter	AUC	T-tests	Log2 FC
CRP	0.78075	$4,29 \times 10^{-23}$	-1.5362
Iron	0.7622	2.28×10^{-14}	0.91002
Ferritin	0.73797	8.62×10^{-13}	-1.219
LDH	0.73539	1.30×10^{-12}	-0.36064
Glyc/SPC	0.72973	2.69×10^{-14}	-0.39473
GlycA	0.71877	1.86×10^{-13}	-0.20232
Glyc	0.71661	2.38×10^{-13}	-0.2007
Phenylalanine	0.71593	8.33×10^{-13}	-0.38819
GFR (CKD-EPI)	0.71557	9.62×10^{-12}	0.23299
Transferrin	0.71542	6.92×10^{-14}	0.26628
Transferrin saturation	0.71485	3.02×10^{-11}	0.68091
TPA1	0,71334	2.77×10^{-11}	0.19877
HDA1	0.71218	2.07×10^{-11}	0.20407
GlycB	0.70758	9.57×10^{-13}	-0.19665
Creatinine	0.70755	1.68×10^{-5}	-0.3557
H4A1	0.70318	4.85×10^{-12}	0.22142
H3FC	0.70271	3.15×10^{-12}	0.43064
Acetoacetic acid	0.69915	9.28×10^{-5}	-1.2853
H4PL	0.68212	5.35×10^{-10}	0.2087
SPC	0.67284	9.47×10^{-8}	0.24121

Table S9. Lists of abbreviations and units

Overview of the used abbreviations for clinical Parameter and Markers from B.I. QUANT-PS and B.I. PACS

Nr.	Name	Extended name	Unit
1	CRP	C-reactive protein	mg/L
2	WBC	White Blood Cells	$x10^9$cells/L
3	Lymph	Lymphocytes	$x10^9$cells/L
4	LDH	Lactate Dehydrogenase	Units/L
5	AST	Aspartate-Aminotransferase	Units/L
6	GGT	Gamma-Glutamyltransferase	Units/L
7	GFR	Glomerular Filtration Rate	mL/min
8	Crea	Creatinine	mg/dL
9	Transf. Sat	Transferrin Saturation	%
10	Beta HB acid	Beta-Hydroxybutyric acid	mmol/L
11	Gln/Glu	Ratio of Glutamine and Glutamic Acid	None
12	Glyc	Glycoprotein	Procedure defined unit
13	SPC	Supramolecular Phospholipid Composite	Procedure defined unit

Overview of the used Abbreviations for the Lipid Profile

Nr.	Name	Extended name	Unit
1	ABA1	Apolipoprotein-B100/Apolipoprotein-A1	none-
2	H1A1	Apolipoprotein-A1 HDL-1	mg/dL
3	H1A2	Apolipoprotein-A2 HDL-1	mg/dL
4	H1CH	Cholesterol HDL-1	mg/dL
5	H1FC	Free Cholesterol HDL-1	mg/dL
6	H1PL	Phospholipids HDL-1	mg/dL
7	H1TG	Triglycerides HDL-1	mg/dL
8	H2A1	Apolipoprotein-A1 HDL-2	mg/dL
9	H2A2	Apolipoprotein-A2 HDL-2	mg/dL
10	H2CH	Cholesterol HDL-2	mg/dL
11	H2FC	Free Cholesterol HDL-2	mg/dL
12	H2PL	Phospholipids HDL-2	mg/dL
13	H2TG	Triglycerides HDL-2	mg/dL
14	H3A1	Apolipoprotein-A1 HDL-3	mg/dL
15	H3A2	Apolipoprotein-A2 HDL-3	mg/dL
16	H3CH	Cholesterol HDL-3	mg/dL
17	H3FC	Free Cholesterol HDL-3	mg/dL

18	H3PL	Phospholipids HDL-3	mg/dL
19	H3TG	Triglycerides HDL-3	mg/dL
20	H4A1	Apolipoprotein-A1 HDL-4	mg/dL
21	H4A2	Apolipoprotein-A2 HDL-4	mg/dL
22	H4CH	Cholesterol HDL-4	mg/dL
23	H4FC	Free Cholesterol HDL-4	mg/dL
24	H4PL	Phospholipids HDL-4	mg/dL
25	H4TG	Triglycerides HDL-4	mg/dL
26	HDA1	HDL-Apolipoprotein-A1	mg/dL
27	HDA2	HDL-Apolipoprotein-A2	mg/dL
28	HDCH	HDL-Cholesterol	mg/dL
29	HDFC	HDL Free Cholesterol	mg/dL
30	HDPL	HDL Phospholipids	mg/dL
31	HDTG	HDL Triglycerides	mg/dL
32	IDAB	IDL-Apolipoprotein-B100	mg/dL
33	IDCH	IDL Cholesterol	mg/dL
34	IDFC	IDL Free Cholesterol	mg/dL
35	IDPL	IDL Phospholipids	mg/dL
36	IDPN	ILDL Particle Number	nmol/L
37	IDTG	IDL Triglycerides	mg/dL
38	L1AB	Apolipoprotein-B100 LDL-1	mg/dL
39	L1CH	Cholesterol LDL-1	mg/dL
40	L1FC	Free Cholesterol LDL-1	mg/dL
41	L1PL	Phospholipids LDL-1	mg/dL
42	L1PN	Particle Number LDL-1	nmol/L
43	L1TG	Triglycerides LDL-1	mg/dL
44	L2AB	Apolipoprotein-B100 LDL-2	mg/dL
45	L2CH	Cholesterol LDL-2	mg/dL
46	L2FC	Free Cholesterol LDL-2	mg/dL
47	L2PL	Phospholipids LDL-2	mg/dL
48	L2PN	Particle Number LDL-2	nmol/L
49	L2TG	Triglycerides LDL-2	mg/dL
50	L3AB	Apolipoprotein-B100 LDL-3	mg/dL
51	L3CH	Cholesterol LDL-3	mg/dL
52	L3FC	Free Cholesterol LDL-3	mg/dL
53	L3PL	Phospholipids LDL-3	mg/dL
54	L3PN	Particle Number LDL-3	nmol/L
55	L3TG	Triglycerides LDL-3	mg/dL
56	L4AB	Apolipoprotein-B100 LDL-4	mg/dL
57	L4CH	Cholesterol LDL-4	mg/dL
58	L4FC	Free Cholesterol LDL-4	mg/dL
59	L4PL	Phospholipids LDL-4	mg/dL
60	L4PN	Particle Number LDL-4	nmol/L
61	L4TG	Triglycerides LDL-4	mg/dL
62	L5AB	Apolipoprotein-B100 LDL-5	mg/dL
63	L5CH	Cholesterol LDL-5	mg/dL

64	L5FC	Free Cholesterol LDL-5	mg/dL
65	L5PL	Phospholipids LDL-5	mg/dL
66	L5PN	Particle Number LDL-5	nmol/L
67	L5TG	Triglycerides LDL-5	mg/dL
68	L6AB	Apolipoprotein-B100 LDL-6	mg/dL
69	L6CH	Cholesterol LDL-6	mg/dL
70	L6FC	Free Cholesterol LDL-6	mg/dL
71	L6PL	Phospholipids LDL-6	mg/dL
72	L6PN	Particle Number LDL-6	nmol/L
73	L6TG	Triglycerides LDL-6	mg/dL
74	LDAB	LDL-Apolipoprotein-B100	mg/dL
75	LDCH	LDL-Cholesterol	mg/dL
76	LDFC	LDL Free Cholesterol	mg/dL
77	LDHD	LDL-cholesterol/HDL-cholesterol	-
78	LDPL	LDL Phospholipids	mg/dL
79	LDPN	LDL Particle Number	nmol/L
80	LDTG	LDL Triglycerides	mg/dL
81	TBPN	Total Particle Number (apolipoprotein-B100 carrying particles)	nmol/L
82	TPA1	Total Plasma Apolipoprotein-A1	mg/dL
83	TPA2	Total Plasma Apolipoprotein-A2	mg/dL
84	TPAB	Total Plasma Apolipoprotein-B100	mg/dL
85	TPCH	Total Plasma Cholesterol	mg/dL
86	TPTG	Total Plasma Triglycerides	mg/dL
87	V1CH	Cholesterol VLDL-1	mg/dL
88	V1FC	Free Cholesterol VLDL-1	mg/dL
89	V1PL	Phospholipids VLDL-1	mg/dL
90	V1TG	Triglycerides VLDL-1	mg/dL
91	V2CH	Cholesterol VLDL-2	mg/dL
92	V2FC	Free Cholesterol VLDL-2	mg/dL
93	V2PL	Phospholipids VLDL-2	mg/dL
94	V2TG	Triglycerides VLDL-2	mg/dL
95	V3CH	Cholesterol VLDL-3	mg/dL
96	V3FC	Free Cholesterol VLDL-3	mg/dL
97	V3PL	Phospholipids VLDL-3	mg/dL
98	V3TG	Triglycerides VLDL-3	mg/dL
99	V4CH	Cholesterol VLDL-4	mg/dL
100	V4FC	Free Cholesterol VLDL-4	mg/dL
101	V4PL	Phospholipids VLDL-4	mg/dL
102	V4TG	Triglycerides VLDL-4	mg/dL
103	V5CH	Cholesterol VLDL-5	mg/dL
104	V5FC	Free Cholesterol VLDL-5	mg/dL
105	V5PL	Phospholipids VLDL-5	mg/dL
106	V5TG	Triglycerides VLDL-5	mg/dL
107	VLAB	VLDL-Apolipoprotein-B100	mg/dL
108	VLCH	VLDL Cholesterol	mg/dL

109	VLFC	VLDL Free Cholesterol	mg/dL
110	VLPL	VLDL Phospholipids	mg/dL
111	VLPN	VLDL Particle Number	nmol/L
112	VLTG	VLDL Triglycerides	mg/dL

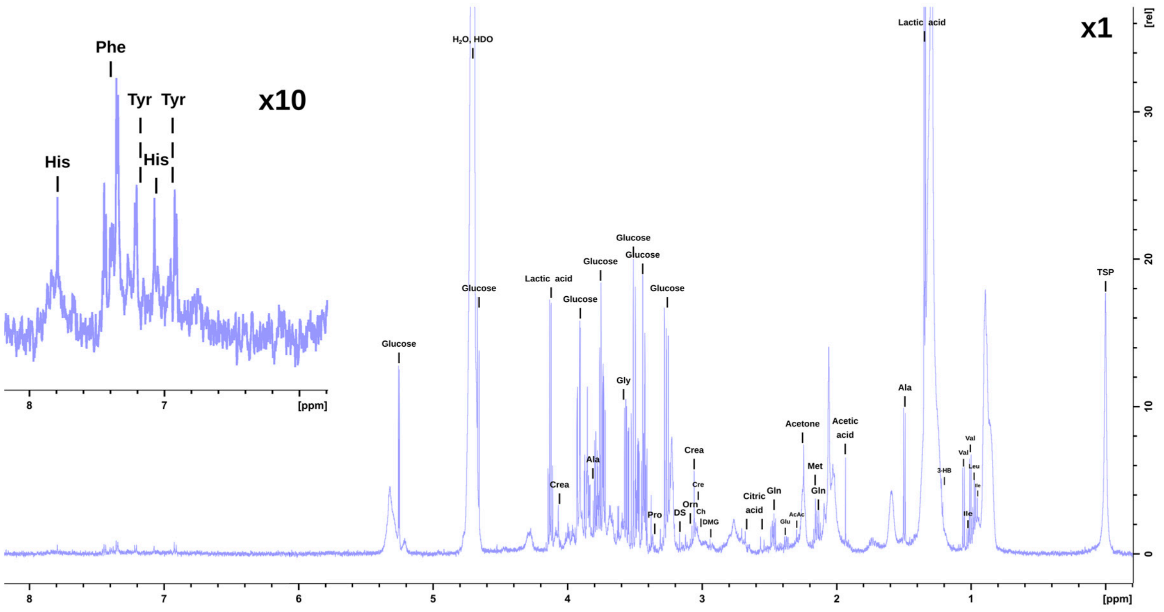

(a)

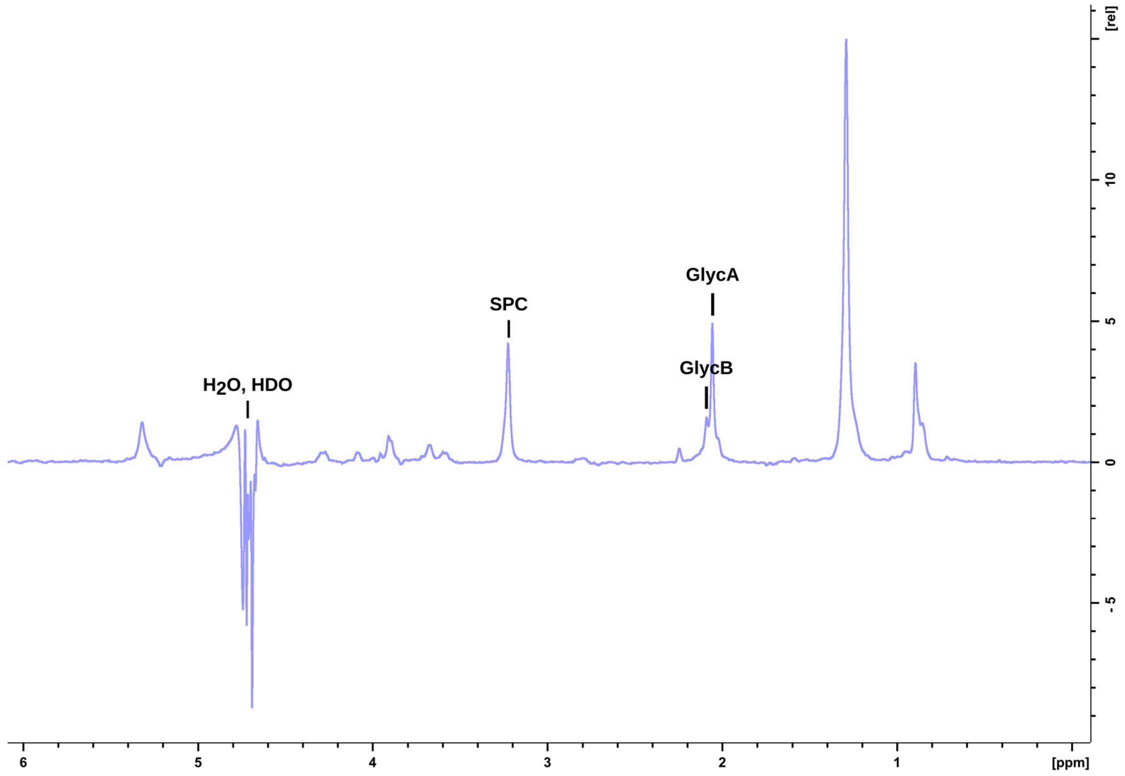

(b)

Figure S1. 1D ^{1}H NMR spectral data of one of the blood analyte solutions used to analyze polar measurable compounds such as amino acids and etc., as well as spectral data providing glycoprotein and SPC levels.
Panel a, The example CPMG spectrum displays quantified metabolites, whereas the aromatic region (from 8.0 till 6.0 ppm) was zoomed in by a factor of 10 times. Used abbreviations: His – histidine, Phe – phenylalanine, Tyr – tyrosine, HDO – semiheavy water, Crea – creatinine, Ala – alanine, Gly – glycine, Pro – Proline, DS – dimethylsulfone, Cre – creatine, Ch – choline, DMG – N,N-dimethylglycine, Gln – glutamine, Glu – glutamic acid, AcAc – acetoactic acid, Met – methionine, 3-HB – 3-hydroxybutyric acid, Val – valine, Ile – isoleucine, Leu – leucine, TSP – a chemical shift indicatior (δ = 0.00 ppm), 3-(trimethylsilyl)propionic2,2,3,3 d4 acid.

Panel b, The example PGPE spectrum displays those parameters that are utilized within the Inflammatory section of the Bruker's PhenoRisk PACS™ RuO* test. Used abbreviations: HDO – semiheavy water, SPC – Supramolecular Phospholipid Composite, GlycB – glycoprotein B, GlycA – glycoprotein A.

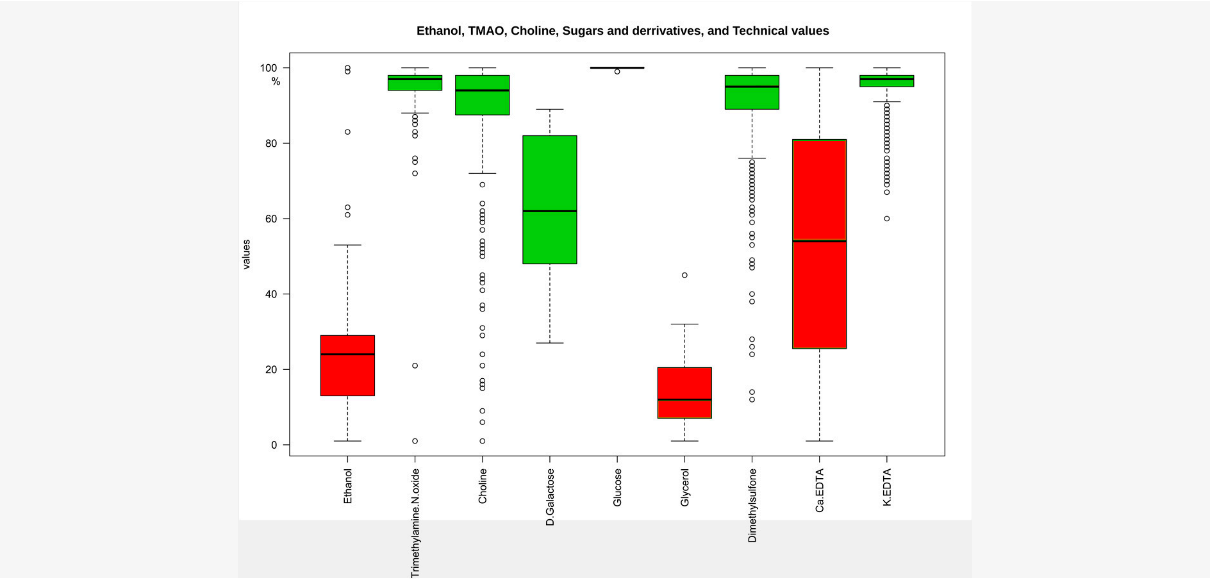

Ethanol, TMAO, Choline, Sugars and derrivatives, and Technical values
values
100 %
80
60
40
20
0
Ethanol
Trimethylamine.N.oxide
Choline
D.Galactose
Glucose
Glycerol
Dimethylsulfone
Ca.EDTA
K.EDTA

(a)

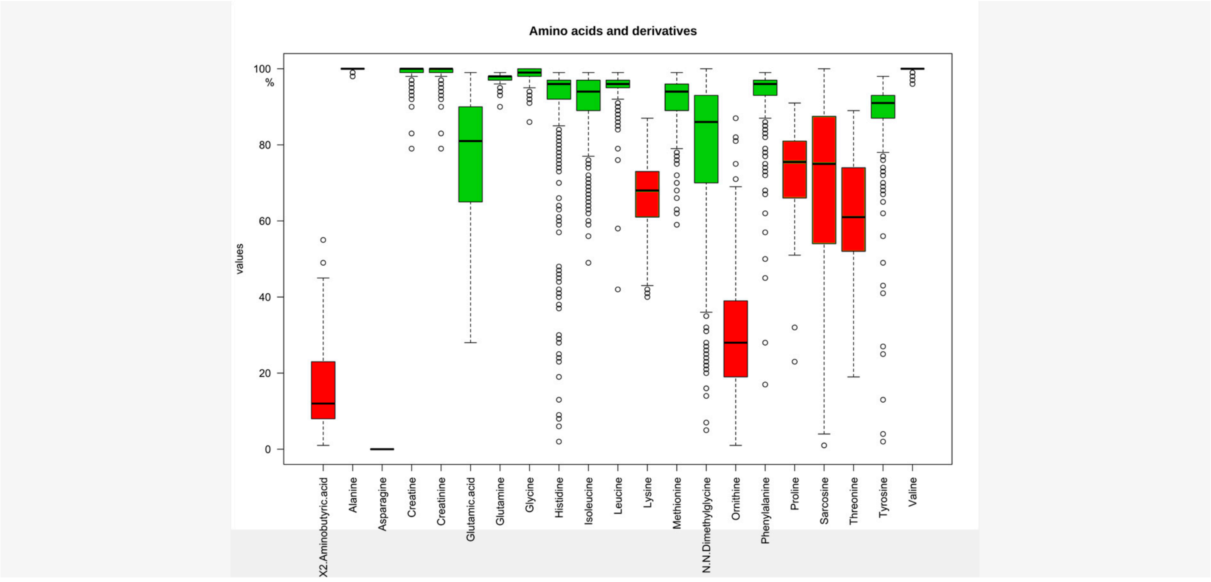

Amino acids and derivatives
values
100 %
80
60
40
20
0
X2.Aminobutyric.acid
Alanine
Asparagine
Creatine
Creatinine
Glutamic.acid
Glutamine
Glycine
Histidine
Isoleucine
Leucine
Lysine
Methionine
N.N.Dimethylglycine
Ornithine
Phenylalanine
Proline
Sarcosine
Threonine
Tyrosine
Valine

(b)

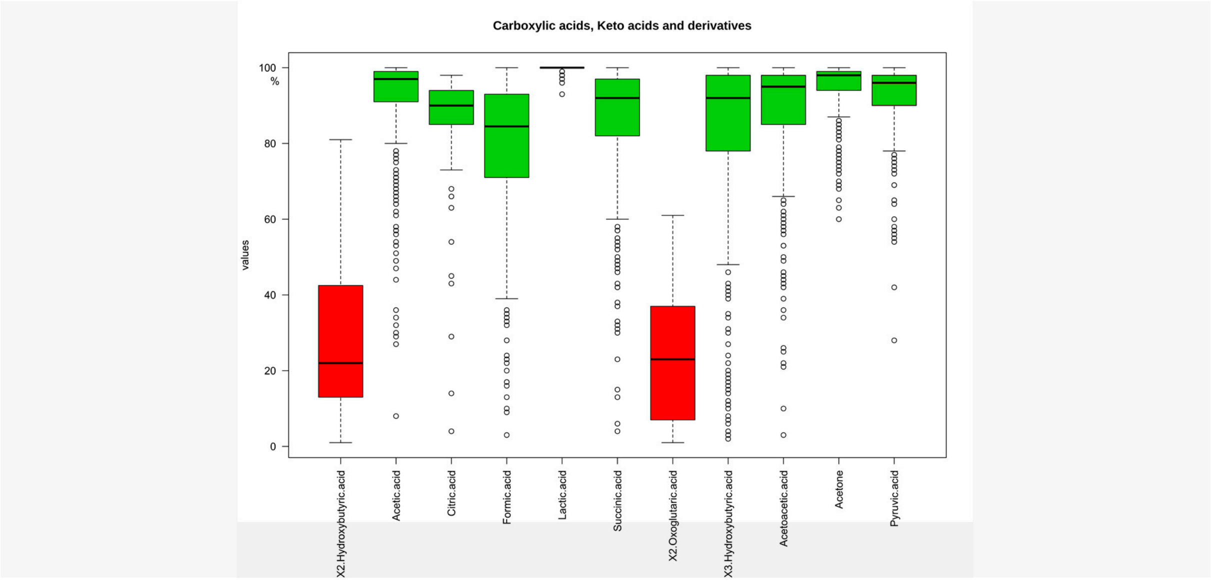

Carboxylic acids, Keto acids and derivatives
values
100 %
80
60
40
20
0
X2.Hydroxybutyric.acid
Acetic.acid
Citric.acid
Formic.acid
Lactic.acid
Succinic.acid
X2.Oxoglutaric.acid
X3.Hydroxybutyric.acid
Acetoacetic.acid
Acetone
Pyruvic.acid

(c)

Figure S2 Overview of the ϱ-values in BI-QUANT-PS™ Analysis Report: multiple box plot panels based on the data provided by the Plasma/Serum B.I.Quant-PS™. The plots represent additional concentration range information (% range) provided in order to evaluate analytical quality of the NMR quantitative data used exclusively for the patients' cohort part of the analysis within the current study, i.e. none of the healthy controls' NMR data had been used within the graphical illustrations. Green box plots represent mean ϱ values >80%, red box plots <80%

Panel a, Data for the ethanol, TMAO, Sugars and EDTA values.

Panel b, Data for the Amino acids and their derrivative compounds.

Panel c, Data for the Carboxylic acids, Keto acids and their derrivative compounds.

PCA

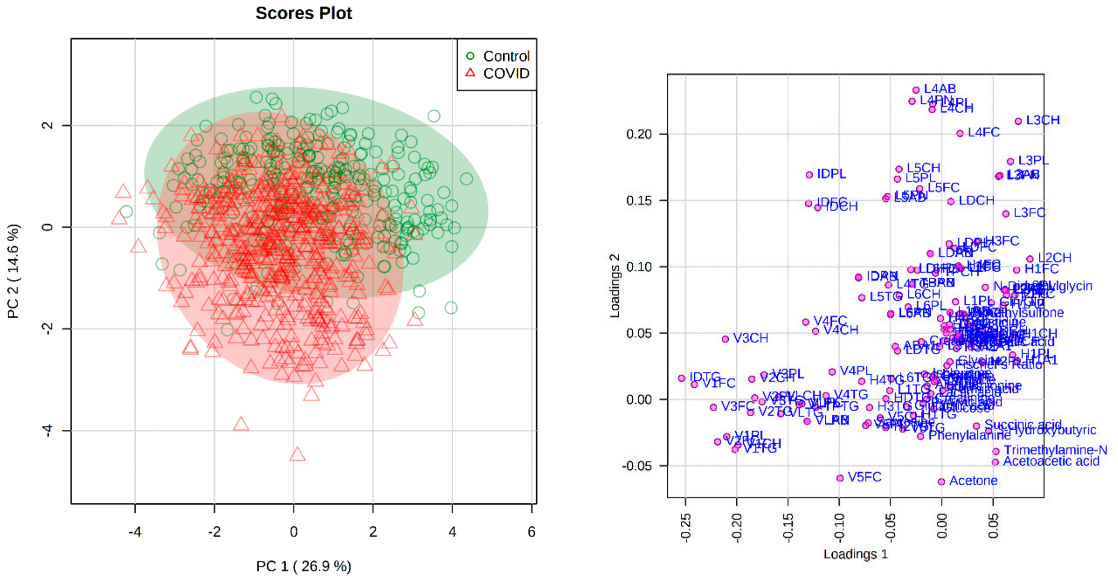

OPLS-DA

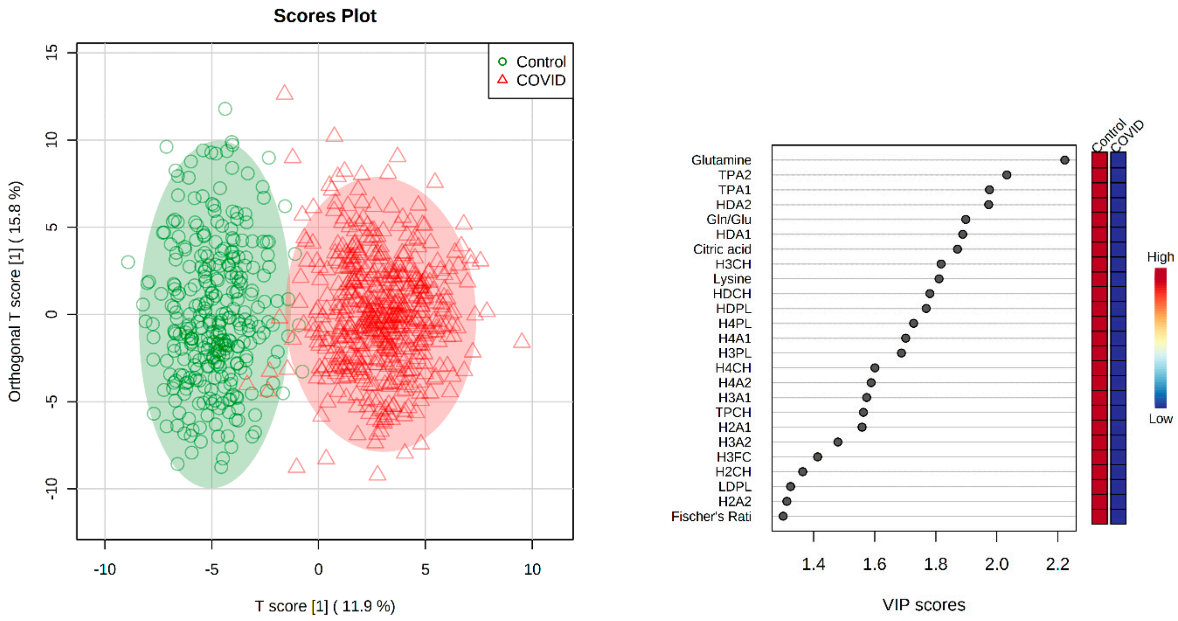

Figure S3. Multivariate Analysis COVID-19 vs. Healthy controls utilizing PCA analysis and OPLS-DA regression analysis

PCA

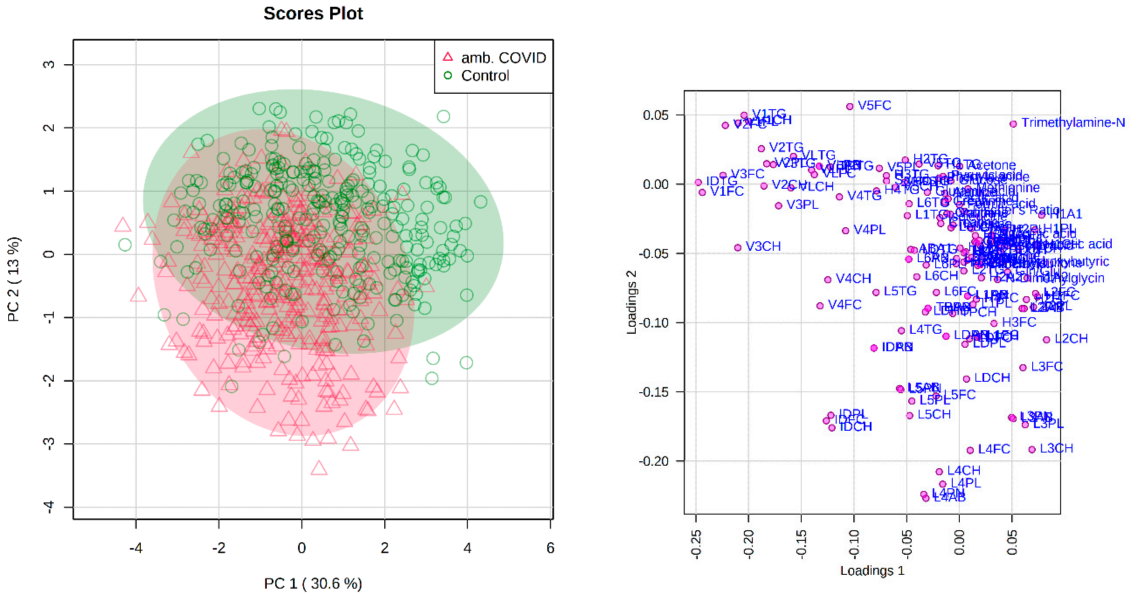

OPLS-DA

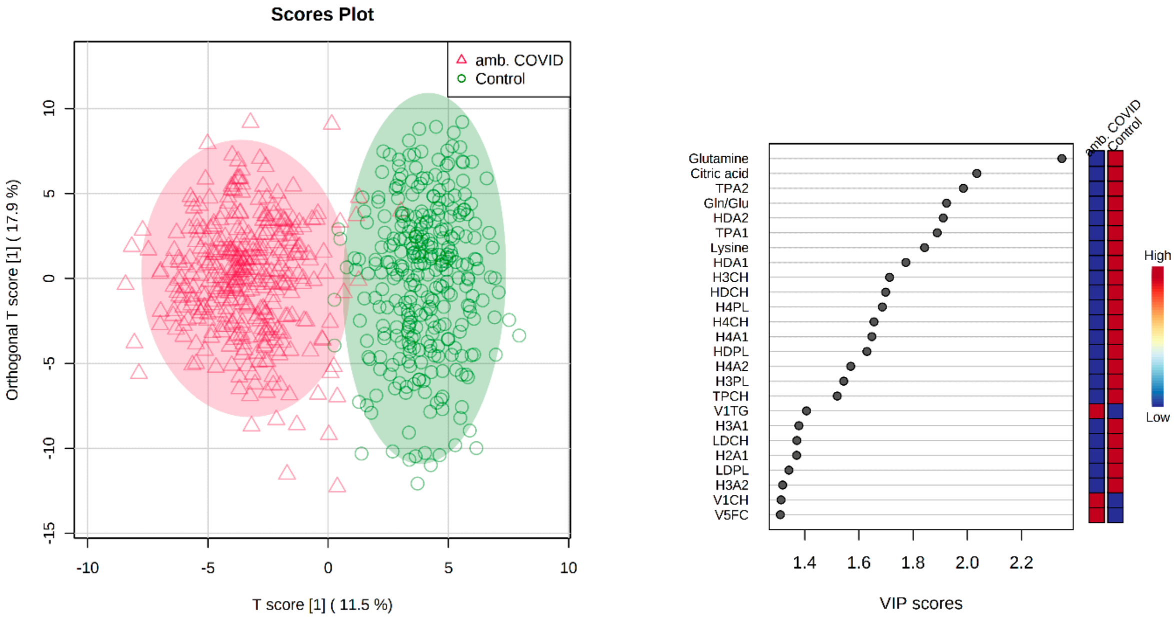

Figure S4. Multivariate Analysis of ambulatory COVID-19 patients vs. Healthy controls utilizing PCA and OPLS-DA regression analysis

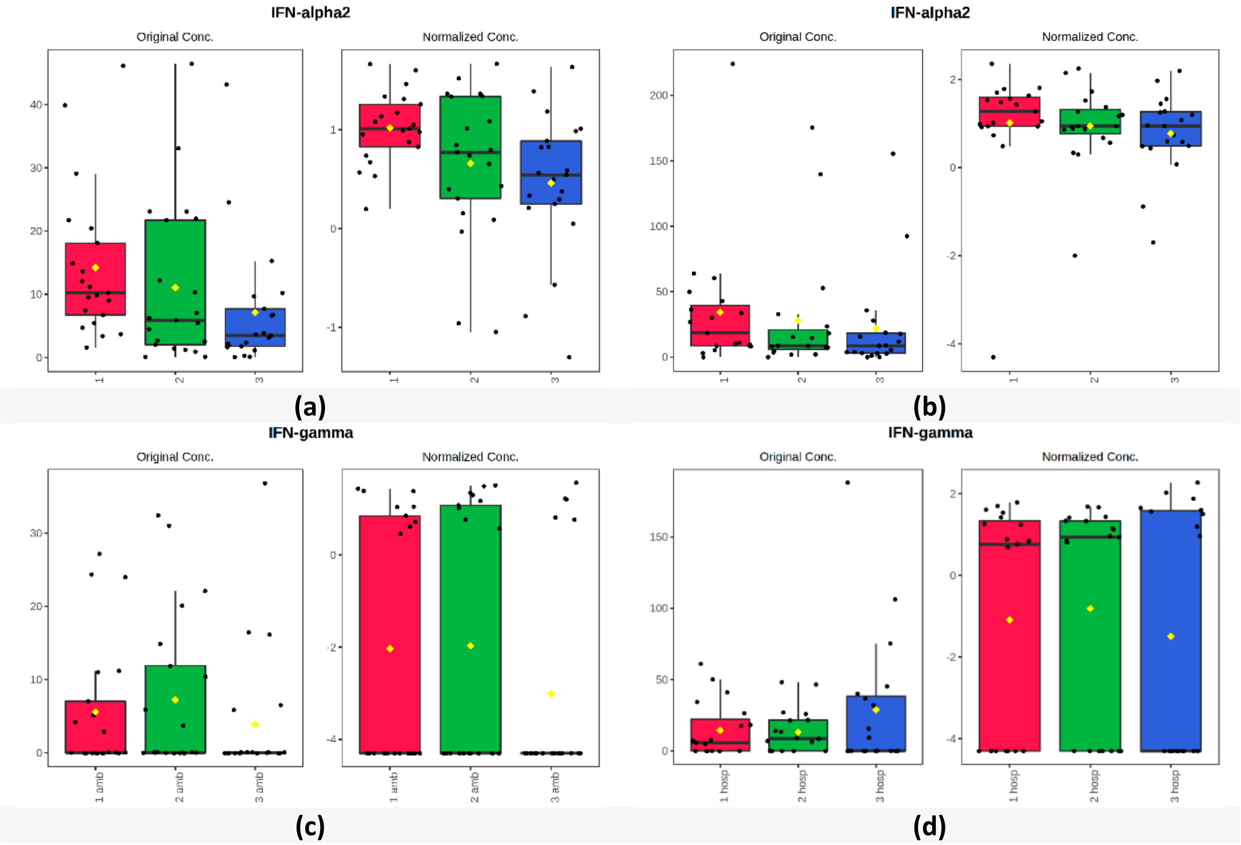

Figure S5. Box plots of cytokine trajectory (the normalization process consisted of logarithmic transformation)

Panel a, IFN-alpha2-levels in individuals who self-cured at home and gave 3 serum samples.
Panel b, IFN-alpha2-levels in hospitalized patients who supplied 3 serum samples.
Panel c, IFN-gamma levels in patients who self-cured at home and supplied 3 serum samples.
Panel d, IFN-gamma in hospitalized patients who donated 3 samples.
